# Less reactive dipoles of diazodicarbonyl compounds in reaction with cycloaliphatic thioketones – First evidence for the 1,3-oxathiole–thiocarbonyl ylide interconversion

**DOI:** 10.3762/bjoc.9.309

**Published:** 2013-12-02

**Authors:** Valerij A Nikolaev, Alexey V Ivanov, Ludmila L Rodina, Grzegorz Mlostoń

**Affiliations:** 1 Saint-Petersburg State University, 198504, Saint Petersburg, University prosp., 26, Russia; 2 University of Łódź, Faculty of Chemistry, Tamka 12, 91-403, Łódź, Poland

**Keywords:** 1,3-dipolar electrocyclization, 1,5-dipolar electrocyclization, 1,3-oxathioles, thiocarbonyl ylides, thiiranes, thioketones

## Abstract

Acyclic diazodicarbonyl compounds react at room temperature with cycloaliphatic thioketones, e.g. 2,2,4,4-tetramethyl-3-thioxocyclobutanе-1-one and adamantanethione, via a cascade process in which the key step is a 1,5-electrocyclization of the intermediate thiocarbonyl ylide leading to tetrasubstituted spirocyclic 1,3-oxathioles. The most reactive diazodicarbonyl compound was diazoacetylacetone. In the case of dimethyl diazomalonate competitive 1,3-electrocyclization yielded the corresponding thiirane at elevated temperature, which after spontaneous desulfurization produced a tetrasubstituted alkene. To explain the observed temperature dependence of the main reaction product type obtained from dimethyl diazomalonate and 2,2,4,4-tetramethyl-3-thioxocyclobutanе-1-one as well as to verify reversibility of the thiocarbonyl ylide and 1,3-oxathiole interconversion, the calculations of the energy profile for the transformation of 1,3-oxathiole to alkene were performed at the DFT PBE1PBE/6-31G(d) level.

## Introduction

Aryl- and alkylsubstituted thioketones exhibit high 1,3-dipolar reactivity towards diazoalkanes, diazoesters and diazoketones [1–4]. Due to their high dipolarophilic reactivity thioketones were given the name ‘superdipolarophiles’ [3–4]. It might be expected that these highly reactive dipolarophiles could also easily react with the deactivated 1,3-dipoles of 2-diazo-1,3-dicarbonyl compounds. Preliminary experiments, however, showed that under standard conditions, a cycloaddition of diazodimedone and dimethyl diazomalonate with thiobenzophenone did not occur [5–6]. On the other hand, recent studies showed that the [3 + 2]-cycloaddition of this thioketone with many diazodicarbonyl compounds takes place, but at room temperature the reaction proceeds very slowly [7–8].

Within the framework of our longstanding research interest in the synthesis of heterocycles by using diazo compounds [4,9–13], we have recently performed a comprehensive study of a variety of reactions of diazodicarbonyl compounds with arylsubstituted (aromatic) thioketones to establish their suitability for the preparation of 1,3-oxathioles and other sulfur-containing heterocycles [7–8]. The main goal of the present study was to investigate the scope and limitations of cycloaddition reactions of 2-diazo-1,3-dicarbonyl compounds with cycloaliphatic thioketones. The study was aimed at (i) the determination of the key directions of these processes in dependence of the type of diazo compound and (ii) the identification of their usefulness for the synthesis of sulfur-containing heterocycles and derived compounds.

## Results and Discussion

**Figure 1:**
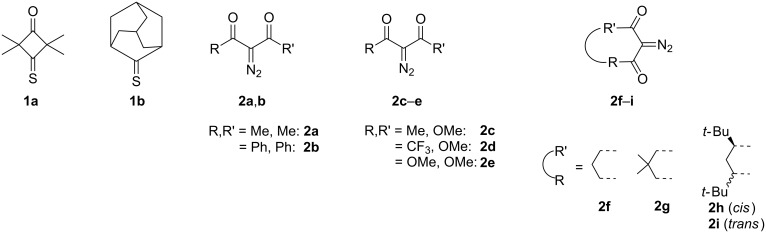
Thioketones **1** and diazodicarbonyl compounds **2**.

Two cycloaliphatic thioketones that were previously studied in similar reactions with diazo compounds [14], were selected as dipolarophiles for the present study, namely 2,2,4,4-tetramethyl-3-thioxocyclobutanе-1-one (**1a**) [15] and adamantane-2-thione (**1b**) [16] (Figure 1). As for the diazo-dipoles, selected diazodicarbonyl compounds **2** with different substitution patterns, including acyclic diazodiketones **2a**–**b**, fluoroalkyl-containing (***F***) and fluorine-free (***H***) diazoketoesters **2c**,**d**, diazomalonic ester **2e**, and carbocyclic diazodiketones **2f**–**i** were tested (Figure 1).

The reactions were carried out either at room temperature or at 80 °C depending on the reactivity and the stability of diazodicarbonyl compound **2** and thioketone **1**. In general, ca. 5% excess of **2** was applied in order to enable a visual determination of the completion of the reaction based on the disappearance of the intensive red or orange color of thioketone **1**. In order to enhance the concentration of the reagents and thereby increase the rate of the reaction, experiments with liquid diazocompounds **2a**,**c–e**,**h** and **i** and thioketone **1a** were carried out under solvent-free conditions. On the other hand, reactions of solid diazocyclohexanedione (**2f**) and diazodimedone (**2g**) as well as all reactions with dibenzoyldiazomethane (**2b**), were performed at rt by using toluene as a solvent.

Unlike the aromatic analogues, thioketone **1а** is completely stable under standard conditions [15]. Therefore, all experiments performed with this thioketone did not require an inert gas atmosphere or other special precautions (Table 1).

**Table 1:** Reactions of diazodiketones **2** with thioketone **1a**.

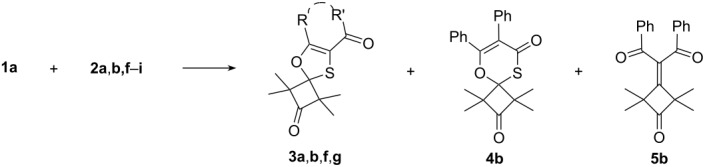				

entry	conditions	yields^a^		

	(temp., time)	**3a**,**b**,**f**,**g**	**4b**	**5b**

1	**2a**, rt, 31 d	79% (88%^b^)	–	–
2	**2b**, rt, 70 d	35% (44%)	–	(6%)
3	**2b**, 80 °C, 3 h	19% (25%)	(17%)	(11%)
4	**2f**, 80 °C, 72 h	7% (14%)	–	–
5	**2g**, 80 °C, 90 h	18% (25%)	–	–
6^c^	**2h**,**i**			

^a^Isolated yields, entries in parentheses refer to yields determined by ^1^H NMR; ^b^combined yield of isolated crystalline product **2a** and the product content in filtrate, determined by ^1^H NMR; ^c^no reaction observed under any of the conditions.

**Figure 2:**
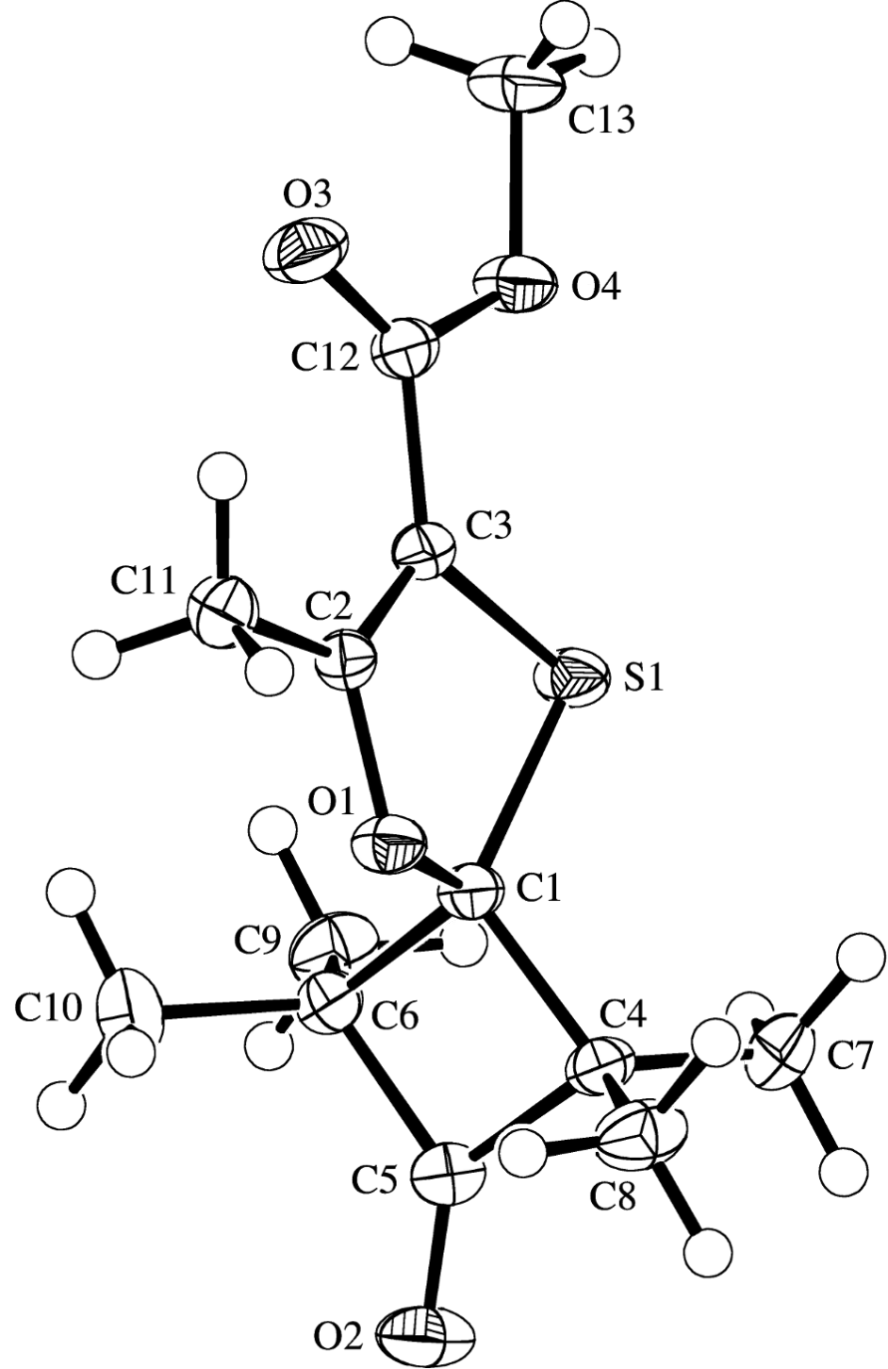
ORTEP plot [17] of the molecular structure of the 1,3-oxathiole **3a** (50% probability ellipsoids; arbitrary numbering of atoms).

It was found that the acyclic ***H***-diazodiketone **2a** reacted even at room temperature with thioketone **1a** to give the spirocyclic 1,3-oxathiole **3a** as the sole reaction product in 79% yield (88% yield was determined by ^1^Н NMR spectroscopy (Table 1, entry 1). After isolation and crystallization from diethyl ether, the postulated structure of **3a** was unambiguously confirmed by X-ray single crystal diffraction analysis (Figure 2).

In the reaction of dibenzoyldiazomethane (**2b**) with thioketone **1a**, performed at rt, the yield of oxathiole **3b** was two times lower (Table 1, entry 2). Increasing the temperature to 80 °C slightly speeded up the process. However, due to the relatively low thermal stability of diazodiketone **2b** [7–8,18], side reactions resulting in the formation of oxathiinone **4b** (17%) and tetrasubstituted olefin **5b** (11%) were observed (Table 1, entry 3).

Diazocyclohexanedione **2f** and diazodimedone **2g** did not react with thioketone **1a** at rt but the experiments performed at 80 °C (72–90 h) gave rise to 1,3-oxathioles **3f**,**g** albeit in rather low yields (14–25%) (Table 1, entries 4 and 5). As for the sterically crowded representatives **2h**–**i**, neither these diazo compounds nor the corresponding acylketenes generated by their thermolysis [18–19] reacted with thioketone **1a** (as proved by ^1^H NMR spectroscopy) (Table 1, entry 6).

Irrespective of the reaction temperature (rt or 80 °C), methyl diazoacetoacetate (**2с**) reacted with thioketone **1a** similarly to diazodiketone **2a**, yielding 1,3-oxathiole **3с** as a sole reaction product in good yields (77–78%) (Table 2, entries 1 and 2). Fluorinated diazoacetoacetate **2d** reacted with thioketone **1a** very slowly both at rt or at 80 °C giving rise to 1,3-oxathiole **3d** in a low yield (24%) (Table 2, entries 3 and 4). In the case of dimethyl 2-diazomalonate (**2e**) and thioketone **1a**, the reaction course strongly depended on the reaction conditions. Thus, in the experiment performed at rt the formation of 1,3-oxathiole **3e** (65%) along with alkene **5e** (18%) was observed (the ratio **3e/5e** ca. 78/22). On the other hand, the reaction of **2e** with **1a** carried out at 80 °C led to olefin **5e** (81%), containing only traces of oxathiole **3e** (**3e/5e** ~1/60) (Table 2, entries 5 and 6).

**Table 2:** Reactions of diazoketoesters **2c**–**e** with thioketone **1a**.

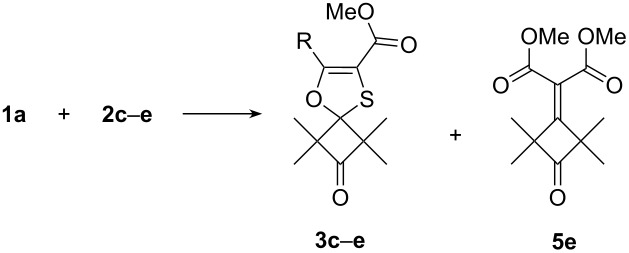			

entry	reaction conditions	yields^a^	
	
	(temp, reaction time)	**3c**–**e**	**5e**

1	**2c**, 20 °C, 60 d	60% (77%)	–
2	**2c**, 80 °C, 20 h	64% (78%)	–
3	**2d**, 20 °C, 60 d	(4%)	–
4	**2d**, 80 °C, 72 h	16% (24%)	–
5	**2e**, 20 °C, 65 d	40% (65%)	13% (18%)
6	**2e**, 80 °C, 20 h	traces	69% (81%)

^a^Isolated yields, entries in parentheses refer to yields determined by ^1^H NMR.

**Scheme 1:**
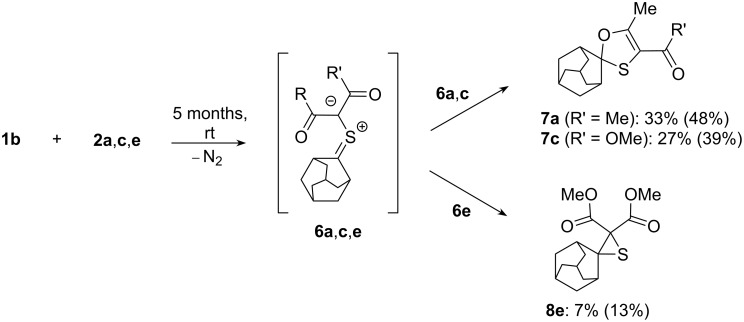
Reaction of diazocarbonyl compounds **2a**,**c**,**e** with adamantane-2-thione (**1b**).

Reactions of diazo compounds **2** with adamantanethione (**1b**) were carried out at room temperature in pentane solution. In comparison with **1a**, **1b** is less stable and tends to undergo dimerization and/or trimerization [16]. For that reason, its reactions were studied by using the most reactive diazodicarbonyl compounds **2a**,**c**,**e** (Scheme 1).

The experiments showed that the reactions of **1b** with diazoacetylacetone **2a** and diazoacetoacetate **2c** performed at rt proceeded similarly as in the case of **1a** yielding spirocyclic tetrasubstituted 1,3-oxathioles **7a**,**c** in moderate yields (39–48%). The thiirane **8e** was isolated as the only product from the mixture obtained after the reaction of diazomalonate **2e** with **1b** in low yield of 13%.

The structures of the isolated compounds **3a**–**g**, **5e**, **7a**,**c**, and **8e** were established by means of spectroscopic methods. Selected characteristic data taken from the ^13^C NMR spectra are summarized in Table 3.

**Table 3:** The key parameters (δ, ppm) of the ^13^C NMR spectra of 1,3-oxathioles **3a**–**e**,**g** and **9a**,**c** (see also [7]).

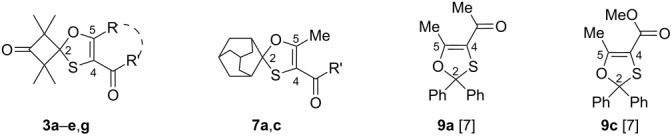					

compound	R,R^1^	C(2)	C(4)	C(5)	CH_3_

**3a**	Me, Me	102.7	111.4	158.0	22.9, 18.3
**3b**	Ph, Ph	102.3	112.1	156.1	23.1, 18.9
**3c**	Me, OMe	102.8	100.7	159.4	22.9, 18.2
**3d**	CF_3_, OMe	105.0	111.5	141.8	22.7, 18.2
**3e**	OMe, OMe	99.3	75.8	159.2	22.5, 18.4
**3g**	–H_2_CCMe_2_CH_2_–	106.8	110.3	166.6	23.1, 18.2
**7a**	Me, Me	105.1	111.5	158.4	–
**7c**	Me, OMe	105.3	100.1	159.7	–
**9a** [7]	Me, Me	101.1	112.6	157.1	–
**9c** [7]	Me, OMe	101.1	101.8	158.4	–

The characteristic signals of the spiroatoms C(2) for the compounds **3a**–**d** and **7a**,**c** were found in a relatively narrow region of the ^13^C NMR spectra (102.3–105.3 ppm; Table 3) similarly to the compounds **9a**,**c** described in literature [7]. The tricyclic analogue **3g** obtained from diazodimedone (**2g**) and thioketone **1a** showed the absorption signal of the C(2)-atom at lower field (106.8 ppm) most likely due to the stronger deshielding effect of the acylcarbonyl group. Nevertheless, the position of the C(2) signal of **3g** does not significantly differ when compared with the data of its bicyclic analogues **3a**–**d**, **7a** and **7c**. For the 1,3-oxathiole **3e** prepared from diazomalonate **2e** the signal of the atom C(2) was found at 99.3 ppm, apparently due to the electron-donating shielding effect of the methoxy group attached to the atom C(5). Similar regularities were also previously reported for 1,3-oxathioles **9a**,**c** isolated after reactions of thiobenzophenone with diazodicarbonyl compounds [7–8].

The chemical shifts of the C(5) atoms in the ^13^C NMR spectra deserve a brief comment. For the series of fluorine-free 1,3-oxathioles **3a**–**c**,**e**, **7a**,**c** and **9a**,**c** [7–8] these signals were detected between 156.0 and 159.5 ppm, while fluoroalkyl-substituted 1,3-oxathiole **3d** displayed the same signal (as a quartet) at higher field (141.8 ppm; Table 3). By contrast, tricyclic derivative **3g** displays a chemical shift of C(5) at a lower field (166.6 ppm), most likely due to the similar reasons as for deshielding effect observed for the atom C(2). However, in the case of С(5) this effect is considerably stronger, since this atom is a vinylogue of the C=O group carbon atom. Analogous effects were also observed in the spectra registered for 1,3-oxathioles derived from thiobenzophenone [7–8]*.* Thus, the collected ^13^C NMR data related to the chemical shifts of the atoms C(2), C(4), and C(5) of the 1,3-oxathioles **3** can be considered as a reliable proof of the postulated structures. Owing to their diastereotopic nature, the four Me groups attached to the cyclobutane ring in 1,3-oxathioles **3a**–**g**, derived from thioketone **1a**, appeared in the ^1^H and ^13^C NMR spectra as two signals (each for 2 Me). This observation can be considered as an additional argument confirming the postulated structure of the 1,3-oxathiole ring [20]. The structure of the thiirane **8e** was established based on the analogy of its main ^1^H and ^13^C NMR signals with a thiirane obtained previously from thiobenzophenone [6,21]. The structure of tetrasubstituted alkene **5e** was confirmed based on a perfect agreement of its ^1^H and ^13^C NMR spectra with literature data [20].

In the ^13^C NMR spectra of the isolated 1,3-oxathioles **7a**,**c** possessing the spiroadamantane fragment, besides the signal of the atom C(2) (at 105.1–105.3 ppm), six other signals attributed to nine C-atoms were observed [2C_t_ (39.9–40.0 ppm.); C_s_ (37.2 ppm); 2C_s_ (35.5 ppm); 2C_s_ (33.2 ppm); C_t_ (26.4 ppm); C_t_ (26.3 ppm)]. The reason for the diminished number of signals is the symmetry plane in the molecules of 1,3-oxathioles **7a**,**c** that goes through the heterocyclic ring. It is noteworthy that the collected results are also in good agreement with the other data reported for the analogous 1,3-oxathioles bearing the spiroadamantane skeleton [5,14].

**Scheme 2:**
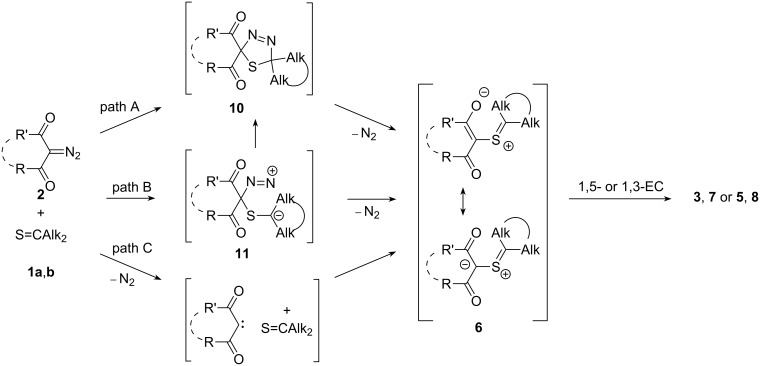
Three possible pathways **A**, **B** and **C** for the formation of 1,3-oxathioles **3**,**7** and thiiranes **5** and **8** from diazodicarbonyl compounds **2** and thioketones **1**.

Hence, the main products of the reactions of thioketones **1a**,**b** with deactivated dipoles of the acyclic 2-diazo-1,3-dicarbonyl compounds **2a**–**g** were 1,3-oxathioles **3** and **7**. The mechanisms of the formation of these compounds can be rationalized by pathways presented in Scheme 2 [4,22].

1) Pathway A implies the initial 1,3-dipolar cycloaddition between the diazo compound **2** and the С=S bond of thioketone **1** giving rise to 1,3,4-thiadiazoline **10**. The latter is usually an unstable intermediate species [1–4] and easily eliminates N_2_ forming the reactive thiocarbonyl ylide **6**. This intermediate, after subsequent 1,5- or 1,3-electrocyclization produces 1,3-oxathioles **3**,**7** or thiiranes **5** and **8**, respectively (Scheme 2, path A) [4–6,22]. In some instances, substituted thiiranes undergo spontaneous desulfurization and convert into tetrasubstituted alkenes [20].

2) Pathway B assumes a stepwise cycloaddition of the diazo-1,3-dipole with the C=S bond leading to the initial formation of the diazonium zwitterion **11**. This step is followed either by the ring closure to give thiadiazoline **10** or by an elimination of nitrogen. Both processes lead to the intermediate thiocarbonyl ylide **6** (Scheme 2, pathway B). The subsequent, competitive intramolecular 1,3- or 1,5-electrocyclizations of ylide **6** will afford the same reaction products as in the case of the pathway A.

3) Finally, the generation of the thiocarbonyl ylide **6** is also possible via an alternative pathway **C**, that implies interaction of a dioxocarbene (formed after thermal decomposition of the diazo compound) with the sulfur atom of the C=S group [4,7–8]. The subsequent intramolecular 1,5-electrocyclization of the same intermediate **6** will lead to 1,3-oxathioles **3**,**7** or thiiranes/alkenes **5** and **8**.

It is known that reactions of diazo compounds with sterically demanding thioketones can give rise to fairly stable 1,3,4-thiadiazolines of type **10**, that can be isolated and do not eliminate nitrogen up to 45 °C [4,23–24]. Hence, the formation of stable 1,3,4-thiadiazolines could also be expected in reactions of the sterically crowded thioketone **1a** with bulky diazodiketones. Based on this assumption, additional attempts were undertaken to isolate or at least identify the proposed intermediate **10** by spectroscopic methods in the reaction of thioketone **1a** with diazoacetylacetone **2a**. However, according to the registered ^1^H and ^13^C NMR spectra, in the reaction mixture +20 °C and −5 °C, solely the signals of the starting materials **1a** or **2a** along with the slowly formed 1,3-oxathiole **3а** were identified. At lower temperatures (refrigerator, below −5 °C) the reaction was essentially ‘frozen’.

According to the computational analysis, the transition states for the formation of 1,3,4-thiadiazoline **10a** via the concerted cycloaddition of the corresponding reagents as well as for its decomposition to thiocarbonyl ylide **6a'** and N_2_ display activation barriers (Δ*G*^#^) of 30.2 and 26.0 kcal·mol^−1^, respectively. On the other hand, the Gibbs free energy changes (Δ*G*) for the same processes are −4.4 and +12.8 kcal·mol^−1^. If diazo compound **2a** reacts with thioketone **2b** via a concerted pathway A, the rate-determining step is the first one because of its higher energy barrier (Δ*G*^#^), and consequently, thiadiazoline **10a** could not be detected in the reaction mixture by means of spectroscopic methods.

Based on the experimental observations one can conclude, that the most likely mechanism of the studied reactions does not involve the formation of a stable 1,3,4-thiadiazoline of type **10**.

Steric hindrance at one terminus of the dipolarophile can promote another, so-called ‘one-bond mechanism’ [23–24] (pathway B), which is also possible in the case of the sterically demanded thioketone **1a**. With the aim to check the validity of this assumption, we attempted to trap the dipolar intermediate **11** with *N*-methylmaleimide [25]. However, from the reaction mixture of thioketone **1a**, diazoacetylacetone **2a** and maleimide (as an intercepting agent) solely 1,3-oxathiole **3a** was isolated in 40% (68%) yield. In the ^1^H NMR spectrum of the crude reaction mixture no additional signals could be observed.

To clarify the possibility of a thermal decomposition of the diazodicarbonyl compounds and the alternative ‘carbene pathway’ (C), the thermal stability of a series of diazo compounds **2** was examined [7–8,17]. The obtained results allow us to conclude that at room temperature the formation of 1,3-oxathioles **3**,**7** occurred not via the initial decomposition of diazo compounds **2** and subsequent generation of thiocarbonyl ylides **6**. Instead, the way via the initial [2 + 3]-cycloaddition of the diazo compounds with the C=S bond (Scheme 2) seems to be the most likely process.

**Scheme 3:**
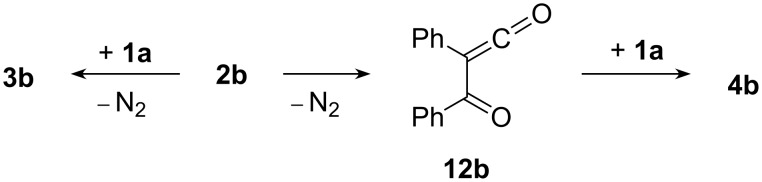
Two competitive transformations of dibenzoyldiazomethane (**2b**) at 80 °С leading to **3b** and **4b**.

The formation of 1,3-oxathiinone **4b** (in addition to 1,3-oxathiole **3b**) in the reaction of diazo compound **2b** with thioketone **1a** at 80 °С is evidently caused by partial thermal decomposition and a Wolff rearrangement of diazodiketone **2b** which gives rise to 2-oxoketene **12b** [7–8,19,26]. The latter reacts with thioketone **1a** in a Diels–Alder reaction, and 1,3-oxathiinone **4b** is formed as a [4 + 2]-cycloadduct (Scheme 3).

The major product of the reaction of diazomalonate **2е** with thioketone **1а** is temperature dependent. Thus, mainly 1,3-oxathiole **3е** is formed at room temperature, while alkene **5е** was obtained as the major product at 80 °С. The formation of the latter can occur via two intermediate steps, i.e. 1,3-electrocyclization of ylide **6** into the corresponding thiirane and subsequent extrusion of the sulfur atom to produce alkene **5е**. An analogous ‘two-fold extrusion process’ was previously observed in catalytic reactions of diazomalonates with thiobenzophenone [6,20].

**Scheme 4:**
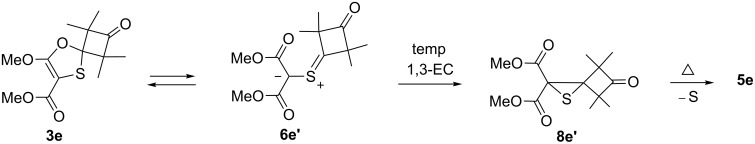
Interconversion of 1,3-oxathiole **3e** and C=S ylide **6e’** accompanied by 1,3-electrocyclization and desulfurization of thiirane **8e’** leading to alkene **5e**.

The most reasonable explanation of the temperature dependence on the outcome of the reaction of diazomalonate **2е** with thioketone **1а** implies the initial formation of 1,3-oxathiole **3е**, which can reversibly convert into thiocarbonyl ylide **6е’**. The latter undergoes cyclization to thiirane **8e’**, which easily eliminates sulfur to furnish alkene **5е** (Scheme 4).

The experiments showed, that at room temperature 1,3-oxathiole **3е** was slowly converted into alkene **5е**, while at 80 °C this process was already completed after a few hours. Thus, the observed process is the first example of a reversible interconversion of a 1,3-oxathiole into the corresponding thiirane via the corresponding thiocarbonyl ylide. Subsequently formed thiirane easily undergoes desulfurization, and finally the respective alkene is formed as an isolable compound. It is well established that the elimination of a sulfur atom S_1_ from thiirane **8e'** has a high positive Δ*G* value (86.6 kcal·mol^−1^). It seems that the desulfurization process occurs via the interaction of two molecules of the thiirane, which results in the formation of an intermediate thiirane *S*-sulfide. The latter undergoes decomposition to the alkene **5e** upon extrusion of disulfur S_2_. Similar mechanisms for the spontaneous desulfurization of oxathiiranes have been reported in a recent publication [27].

**Figure 3:**
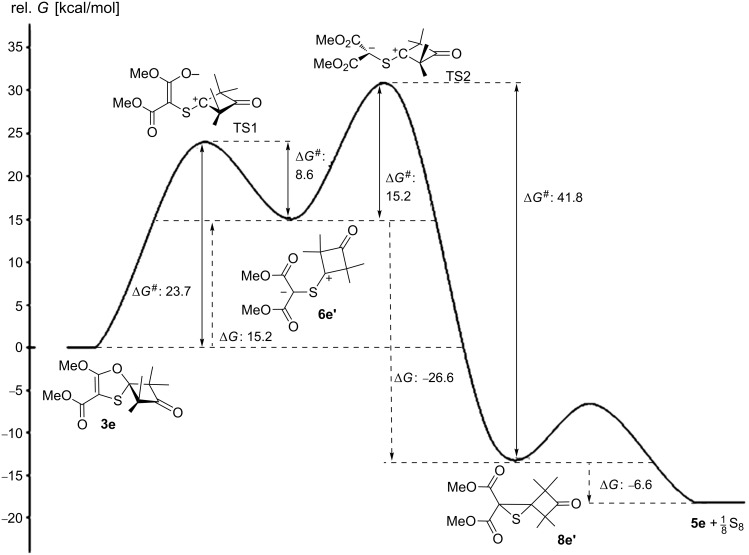
Energy profile for the transformation of 1,3-oxathiole **3e** to alkene **5e**. Relative free energies (kcal·mol^−1^, 298 K) computed at the DFT PBE1PBE/6-31G(d) level.

In order to verify the observed reversibility of the thiocarbonyl ylide **6e'** and oxathiole **3e** interconversion and to explain the observed dependence in the formation of the main reaction product type from the temperature, the computations were performed at the DFT PBE1PBE/6-31G(d) level of the energy profile for the transformation of 1,3-oxathiole **3e** to alkene **5e** (Figure 3, Supporting Information File 1, Table S1).

According to the computations, the transition states for the electrocyclizations of the initially formed thiocarbonyl ylide **6e'** to 1,3-oxathiole **3e** and to thiirane **8e'** display activation barriers (Δ*G*^#^) of 8.6 and 15.2 kcal·mol^−1^, while the Gibbs free energy changes (Δ*G*) for the same processes are 15.2 and 26.6 kcal·mol^−1^, respectively (see Supporting Information File 1, Table S1). Based on these data domination of 1,5-electrocyclization of C=S-ylide **6e'** at room temperature can be apparently explained by the rather high difference (6.6 kcal·mol^−1^) between transition states energy levels TS1 and TS2. At higher temperatures energetically more favorable thiirane **8e'** becomes the main 1,3-electrocyclization product of thiocarbonyl ylide **6e'**. The succeeding decomposition of thiirane **8e'** produces alkene **5e** and sulfur with evolution of 6.6 kcal·mol^−1^. Thus, the reversibility of the thiocarbonyl ylide **6e'** and 1,3-oxathiole **3e** interconversion in this process most likely results from a relatively low activation barrier (26.6 kcal·mol^−1^). It is slowly overcoming even at room temperature giving rise to a gradual accumulation of alkene **5e** during the storage of 1,3-oxathiole **3e** in sealed ampoule.

## Conclusion

We established that acyclic diazodicarbonyl compounds **2a**–**d** react at room temperature with cycloaliphatic thioketones **1a,b** via a cascade process, involving either a concerted or a stepwise cycloaddition of the diazo 1,3-dipole to the C=S bond of thioketone, an elimination of the nitrogen from an intermediate 1,3,4-thiadiazoline or a diazonium zwitterion. Next, electrocyclization of the transient thiocarbonyl ylide leads to spirocyclic 1,3-oxathioles in yields of up to 88%. In similar reactions of diazomalonate **2e**, a competitive process of 1,3-dipolar electrocyclization is observed. It led to the formation of a thiirane derivative, which after subsequent desulfurization is converted into the corresponding tetrasubstituted alkene. At an elevated temperature (80 °С) the process is shifted in this direction, evidently due to reversible interconversion of an intermediate thiocarbonyl ylide and the corresponding 1,3-oxathiole.

Upon increasing the reaction temperature, the rate of the [2 + 3]-cycloaddition of the diazodicarbonyl compounds notably increases, but in the case of diazodiketone **2b** simultaneous thermolysis followed by the Wolff rearrangement occurs. The in situ formed 2-oxoketene reacts as a diene with the C=S bond yielding 1,3-oxathiinone derivative **4b**. The most reactive among the acyclic diazodicarbonyl compounds in our study was diazoacetylacetone **2a**. As for the thioketones reactivity, in line with the expectations, both cycloaliphatic representatives, i.e., 2,2,4,4-tetramethyl-3-thioxocyclobutan-1-one (**1a**) and adamantanethione (**1b**), were found to be less reactive than aromatic thiobenzophenone. However, they are reactive enough to enter reactions with most of the studied diazocarbonyl compounds already at room temperature or upon heating to 80 °C.

## Experimental

**General information:** ^1^H and ^13^C NMR spectra were measured with Bruker 300 and Bruker BioSpin spectrometers, with working frequencies of 300 and 600 MHz for ^1^H NMR and 75.47 and 150.94 MHz for ^13^C NMR spectra, respectively. Solutions were prepared in CDCl_3_ with an internal standard of Me_4_Si (δ, ppm). *J* values are given in Hz. IR spectra were registered by using Perkin-Elmer "Spectrum BXII" instrument as KBr pellets. UV spectra were obtained by using a Shimadzu UV-1800 instrument in EtOH solution. Mass spectra were determined by electrospray ionization with a Bruker micrOTOF spectrometer. Quantitative analysis of the reaction mixtures was performed on the base of the registered ^1^H NMR spectra by using a weighed amount of 1,1,2,2-tetrachloroethane as an internal ‘concentration standard’, prior to the separation of crude reaction mixtures by chromatography or recrystallization. Neutral silicagel L 40/100 (Woelm Pharma) was used for column chromatography. Reaction monitoring and *R*_f_ measurements were performed at Silufol UV-254 (Kavalier, ČSSR) plates. Single crystal X-ray data were collected with a Bruker SMART CCD diffractometer (MoK_α_ radiation, λ = 0.71073 Å, graphite monochromator).

Diazodicarbonyl compounds **2a**–**c** were prepared from the corresponding 1,3-dicarbonyl compounds and arenesulfonyl azides by diazo-transfer reactions [18]. Thioketones **1a**,**b** were prepared from the corresponding ketones by known procedures [15–16].

**General procedure for the reaction of diazodicarbonyl compounds with thioketone 1a:** A mixture of diazodicarbonyl compound **2a**–**i** (1.05 equiv) with thioketone **1a** (1.00 equiv) was allowed to stand in a tightly closed flask at the corresponding temperature (room temperature or 80 °C oil bath) for a particular time. The reaction completion was estimated by the disappearance of the thioketone color in the reaction mixture. The obtained mixture was subjected to ^1^H NMR analysis, and the reaction products were isolated by crystallization, column chromatography or preparative thin-layer chromatography.

**General procedure for the reaction of diazodicarbonyl compounds 2a,c,e with adamantanethione 1b**: A solution of diazo compound **2** (2.1 mmol, 1.05 equiv) and thioketone **1b** (2.0 mmol, 333 mg, 1.00 equiv) in pentane (0.3–0.5 mL) was held in a tightly closed flask at room temperature over 5 months. To remove adamantanethione trimer, a portion of dichloromethane (3–5 mL) was added to the reaction mixture. The insoluble precipitate was separated by filtration and washed with dichloromethane (2 × 1–2 mL). The solvents from the filtrate were evaporated and the reaction products from the residue were isolated by recrystallization or chromatography.

**X-ray crystal-structure determination of 3a.** All measurements were performed on a Nonius KappaCCD area-detector diffractometer [28] by using graphite-monochromated Mo K_α_ radiation (λ 0.71073 Å) and an Oxford Cryosystems Cryostream 700 cooler. The view of the molecule is shown in Figure 2. Data reduction was performed with HKL Denzo and Scalepack [29]. The intensities were corrected for Lorentz and polarization effects, but not for absorption. Equivalent reflections were merged. The structure was solved by direct methods with SHELXS97 [30], which revealed the positions of all non-H atoms. The non-H atoms were refined anisotropically. The hydroxy-H atom was placed in the position indicated by a difference electron density map, and its position was refined together with an isotropic displacement parameter. All remaining H atoms were placed in geometrically calculated positions and refined by using a riding model where each H atom was assigned a fixed isotropic displacement parameter with a value equal to 1.2 *U*_eq_ of its parent C atom (1.5 *U*_eq_ for the methyl groups). The refinement of the structure was carried out on *F*^2^ by using full-matrix least-square procedures, which minimized the function Σw(*F*_o_^2^ – *F*_c_^2^)^2^. A correction for secondary extinction was applied [31]. One reflection, whose intensity was considered to be an extreme outlier, was omitted from the final refinement. Neutral atom scattering factors for non-H atoms were taken from [32], and the scattering factors for H atoms were taken from [33]. Anomalous dispersion effects were included in *F*_c_ [34], the values for *f'* and *f"* originate from [35]. The values of the mass attenuation coefficients were taken from [36]. All calculations were performed by using the SHELXL97 program [30].

**Computational details.** All calculations were performed with the PBE1PBE density functional method [37] by using the Gaussian suite of quantum chemical programs. Geometry optimizations of intermediates, transition states, reactants, and products in the gas phase were performed at the PBE1PBE/6-31G(d) level by using Gaussian 09 [38]. Stationary points on the respective potential energy surfaces were characterized at the same level of theory by evaluating the corresponding Hessian indices. Careful verification of the unique imaginary frequencies for transition states was carried out to check whether the frequency indeed pertains to the desired reaction coordinate. Intrinsic reaction coordinates (IRC) were calculated to authenticate all transition states. Computed geometries of compounds and transition states and their total energies are available in Supporting Information File 2.

## Supporting Information

File 1 Experimental details for the preparation of the compounds **3a–g**, **5e**, **7a,c**, **8e**, their spectroscopic and analytical data, and ^1^H and ^13^C NMR spectra.

File 2 Details of computational studies: Cartesian coordinates, computed geometries of compounds, transition states, and computed total energies.

## References

[1] Huisgen R, Li X, Giera H, Langhals E (2001). Helv Chim Acta.

[2] Huisgen R, Fisera L, Giera H, Sustmann R (1995). J Am Chem Soc.

[3] Huisgen R, Langhals E (1989). Tetrahedron Lett.

[4] Mlostoń G, Heimgartner H, Padwa A, Pearson J (2003). Thiocarbonyl Ylides. Synthetic Application of 1,3-Dipolar Cycloaddition Chemistry Toward Heterocycles and Natural Products.

[5] Kelmendi B, Mlostoń G, Heimgartner H (2000). Heterocycles.

[6] Mlostoń G, Heimgartner H (1996). Helv Chim Acta.

[7] Nikolaev V A, Ivanov A V, Shakhmin A A, Sieler J, Rodina L L (2012). Tetrahedron Lett.

[8] Nikolaev V A, Ivanov A V, Shakhmin A A, Schulze B, Rodina L L (2011). Russ J Org Chem.

[9] Rodina L L, Medvedev Yu Yu, Moroz P N, Nikolaev V A (2012). Russ J Org Chem.

[10] Supurgibekov M B, Zakharova V M, Sieler J, Nikolaev V A (2011). Tetrahedron Lett.

[11] Nikolaev V V, Schulze B, Heimgartner H, Nikolaev V A (2007). Heterocycles.

[12] Nikolaev V V, Heimgartner H, Linden A, Krylov I S, Nikolaev V A (2006). Eur J Org Chem.

[13] Nikolaev V V, Hennig L, Sieler J, Rodina L L, Schulze B, Nikolaev V A (2005). Org Biomol Chem.

[14] Kägi M, Linden A, Mlostoń G, Heimgartner H (1998). Helv Chim Acta.

[15] Heimgartner H, Mlostoń G (2004). 2,2,4,4-Tetramethylcyclobutane-1-one-3-thione. e-EROS, Encyclopedia of Reagents for Organic Synthesis.

[16] Heimgartner H, Mlostoń G, Romański J (2005). Adamantanethione. e-EROS, Encyclopedia of Reagents for Organic Synthesis.

[17] Johnson C K (1976). ORTEP II, Report ORNL-5138.

[18] Regitz M, Maas G (1986). Diazo Compounds. Properties and Synthesis.

[19] Tidwell T T (2006). Ketenes II.

[20] Mlostoń G, Romański J, Heimgartner H (2002). Pol J Chem.

[R21] 21The ^1^H and ^13^C NMR spectra of the thiirane derived from thiobenzophenone and diazomalonic ester [7] are available in the Supporting Information File 1.

[22] Mlostoń G, Heimgartner H (2011). Curr Org Chem.

[23] Huisgen R, Penelle J, Mlostoń G, Padias A B, Hall H K (1992). J Am Chem Soc.

[24] Mlostoń G, Petit M, Linden A, Heimgartner H (1994). Helv Chim Acta.

[25] Xingya L, Huisgen R (1983). Tetrahedron Lett.

[26] Wentrup C, Heilmayer W, Kollenz G (1994). Synthesis.

[27] Reisenauer H P, Mlostoń G, Romański J, Schreiner P R (2011). Eur J Org Chem.

[28] (1999). KappaCCD Collect Software.

[29] Otwinowski Z, Minor W, Carter C W, Sweet R M (1997). Processing of X-ray diffraction data collected in oscillation mode. Macromolecular Crystallography, Part A.

[30] Sheldrick G M (2008). Acta Crystallogr, Sect A: Found Crystallogr.

[R31] 31CCDC-946347 contains the supplementary crystallographic data for this paper. These data can be obtained free of charge from the Cambridge Crystallographic Data Centre, via http://www.ccdc.cam.ac.uk/data_request/cif.

[32] Maslen E N, Fox G A, O'Keefe M A, Wilson A J C (1992). Table 6.1.1.1. International Tables for Crystallography.

[33] Stewart R F, Davidson E R, Simpson W T (1965). J Chem Phys.

[34] Ibers J A, Hamilton W C (1964). Acta Crystallogr.

[35] Creagh D C, McAuley W J, Wilson A J C (1992). Table 4.2.6.8. International Tables for Crystallography.

[36] Creagh D C, Hubbell J H, Wilson A J C (1992). Table 4.2.4.3. International Tables for Crystallography.

[37] Perdew J P, Burke K, Ernzerhof M (1996). Phys Rev Lett.

[38] (2009). Gaussian 09.

