# Supplementary material for: Less reactive dipoles of diazodicarbonyl compounds in reaction with cycloaliphatic thioketones – First evidence for the 1,3-oxathiole–thiocarbonyl ylide interconversion
Source: Beilstein J Org Chem. 2013 Dec 2;9:2751–61. doi: 10.3762/bjoc.9.309 (PMC3869254; doi:10.3762/bjoc.9.309)

Supporting Information

for

Less reactive dipoles of diazodicarbonyl compounds in reaction with cycloaliphatic thioketones – First evidence for the 1,3-oxathiole–thiocarbonyl ylide interconversion

Valerij A. Nikolaev*[1], Alexey V. Ivanov[1], Ludmila L. Rodina[1] and Grzegorz Mlostoń*[2]

Address: [1]Saint-Petersburg State University, 198504, Saint Petersburg, University prosp., 26, Russia and [2]University of Łódź, Faculty of Chemistry, Tamka 12, 91-403, Łódź, Poland

E-mail: Grzegorz Mlostoń - gmloston@uni.lodz.pl and Valerij A. Nikolaev - vnikola@VN6646.spb.edu

*Corresponding author

Experimental details for the preparation of the compounds **3a–g**, **5e**, **7a,c**, **8e**, their spectroscopic and analytical data and ^{1}H and ^{13}C NMR spectra.

Experimental

2. Reactions of diazodicarbonyl compounds with thioketone 1a:

7-Acetyl-1,1,3,3,6-pentamethyl-5-oxa-8-thiaspiro[3.4]oct-6-ene-2-one (3a): (a) The reaction was performed according to the general procedure at room temperature with thioketone **1a** (328 mg, 2.1 mmol) in diazodiketone **2a** (278 mg, 2.2 mmol) for 31 days. The precipitated colorless crystals were separated by filtration, washed with cold pentane and recrystallized from diethyl ether. An additional crop of crystalline material was isolated from the filtrate by removing the solvents in vacuo and recrystallization of the obtained residue from diethyl ether. Total yield of 1,3-oxathiole **3a**: 422 mg (1.67 mmol) [79% (88%; which is the combined yield of isolated crystalline product **2a** and the content of the product in the filtrate as determined by ^{1}H NMR using a defined amount of 1,2-dichloroethane as a concentration standard)].

(b) A solution of thioketone **1a** (156 mg, 1.0 mmol) in diazodiketone **2a** (252 mg, 2.0 mmol) was kept at 5 °C for 50 days. The precipitated crystals were separated from the solution, washed with cold pentane and dried. Yield of 1,3-oxathiole **3a**: 115 mg (0.46 mmol), 46% (62%).

(c) A solution of thioketone **1a** (156 mg, 1.0 mmol) and *N*-methylmaleimide (111 mg, 1 mmol) in diazodiketone **2a** (126 mg, 1.0 mmol) was heated in an oil bath at 40 °C for 100 h and then 3 mL of diethyl ether were added to the reaction mixture. Slightly soluble in diethyl ether, *N*-methylmaleimide was separated (106 mg, 95%). Subsequently, the residue was recrystallized from Et_2O to give 1,3-oxathiole **3a** in 40% (68%) yield (101 mg, 0.40 mmol). Attention: no formation of any adduct with *N*-methylmaleimide was observed by ^{1}H NMR spectroscopy.

(d) A solution of thioketone **1a** (10.9 mg, 0.07 mmol) and diazodiketone **2a** (126 mg, 1.0 mmol) in 0.6 mL $CDCl_3$ was placed in a NMR tube and allowed to stand at –5 °C over 6 months. No other signals, besides related to starting compounds, were identified in the ^{1}H NMR spectra thereafter.

3a: colourless solid. M.p. 113–115 °C. R_f = 0.40 (petroleum ether/diethyl ether, 2:1). ^{1}H NMR (600 MHz, $CDCl_3$): δ = 2.30, 2.29 (2 s, 6 H, 2CH_3C=), 1.30, 1.24 (2 s, 12 H, 2(CH_3)$_2$C) ppm. ^{13}C NMR (151 MHz, $CDCl_3$): δ = 218.5 (*C2*=O), 191.3 (CH_3-*C*=O), 158.0 (O-*C*=C), 111.4 (O-C=*C*), 102.7 (*C4*), 66.7 (2*C*Me_2), 30.4 (*C*H_3C=O), 22.9, 18.3 (4*C*H_3), 15.0 (*C*H_3C=C) ppm. IR (KBr): $\tilde{\nu}_{max}$ (I_{rel}) = 3331 (8), 2978 (30), 2872 (21), 1779 (77), 1673 (82), 1588 (100), 1455 (52), 1363 (66), 1263 (70), 1062 (86), 904 (48), 648 (52), 630 (39), 609 (32), 527 (14) cm^{-1} (%). UV (EtOH): λ_{max} (lg ε) = 211 (3.73), 276

(3.02), 335 (3.54) nm. HRMS (ESI): calcd. for $C_{13}H_{19}O_3S[M+H]^+$ 255.1049; found 255.1060.

Crystal data for **3a**: $C_{13}H_{18}O_4S$, M = 270.34, monoclinic, a = 10.2399(1) Å, b = 10.2480(1) Å, c = 13.4410(2) Å, α = 90.00°, β = 98.9277(7)°, γ = 90.00°, V = 1393.39(3) Å^3, T = 160(1) K, space group $P2_1/c$, Z = 4, μ(MoKα) 0.236 [mm^{-1}], 35656 reflections measured, 4069 independent reflections (R_{int} = 0.038). R_1 = 3592 ($I > 2\sigma(I)$);. $wR(F^2)$ = 0.0935 (all data); GOOF(F^2) = 1.028.

7-Benzoyl-1,1,3,3,-tetramethyl-6-phenyl-5-oxa-8-thiaspiro[3.4]oct-6-ene-2-one (3b): (a) Obtained according to general procedure from thioketone **1a** (312 mg, 2.0 mmol) and diazodiketone **2b** (526 mg, 2.1 mmol) in anhydrous toluene (5 mL), that was kept at 18–23 °C for 70 days. The solvent was removed in vacuo (2 mbar/rt) and the resultant residue was recrystallized from methanol to give 264 mg (0.70 mmol) of oxathiole **3a** [35% (44%)].

(b) A solution of thioketone **1a** (156 mg, 1.0 mmol) and diazodiketone **2b** (263 mg, 1.05 mmol) in 5 mL of toluene was kept at 80 °C for 3 h. The solvent was removed in vacuo (2 mbar/80 °C) and the residue was separated using preparative TLC (eluent: dichloromethane) followed by recrystallization from methanol to furnish oxathiole **3b** in 19% (25%) yield (72 mg, 0.19 mmol). In the reaction mixture of this experiment two more compounds were identified using ^{1}H NMR spectroscopy, which by analogy with the literature data were assigned to the structures of oxathiinone **4b** (signals of Me groups at 1.56 and 1.46 ppm) and alkene **5b** (at 1.36 ppm). However isolation of these products in the pure state using silica gel chromatography have not been successful, apparently due to their instability under these conditions.

3b: yellow solid. M.p. 123–125 °C (with decomposition). R_f = 0.42 (dichloromethane). ^{1}H NMR (600 MHz, $CDCl_3$): δ = 7.54-7.52 (m, 2 H, Ph*H*), 7.33-7.30 (m, 1 H, Ph*H*), 7.28-7.26 (m, 2 H, Ph*H*), 7.23-7.20 (m, 1 H, Ph*H*), 1.43, 1.40 (2 s, 12 H, $2(CH_3)_2C$) ppm. ^{13}C NMR (151 MHz, $CDCl_3$): δ = 218.5 (*C2*=O), 190.1 (CH_3-*C*=O), 156.1 (O-*C*=C), 137.5 (1C, Ph*C*), 132.4 (1C, Ph*C*), 130.5 (1C, Ph*C*), 129.4 (2C, Ph*C*), 129.1 (2C, Ph*C*), 128.9 (1C, Ph*C*), 128.1 (2C, Ph*C*), 128.1 (2C, Ph*C*), 112.1 (O-C=*C*), 102.3 (*C4*), 67.0 (2*C*Me_2), 23.1, 18.9 (4*C*H_3) ppm. IR (KBr): $\tilde{\nu}_{max}$ (I_{rel}) = 3065 (15), 2963 (41), 1778 (100), 1628 (100), 1587 (90), 1566 (82), 1490 (48), 1461 (65), 1326 (87), 1139 (59), 1049 (76), 884 (58), 717 (86), 694 (96) cm^{-1} (%). UV (EtOH): λ_{max} (lg ε) = 248 (4.20), 390 (3.70) nm. HRMS (ESI): calcd. for $C_{23}H_{23}O_3S[M+H]^+$ 379.1362; found 379.1369.

Methyl 1,1,3,3,6-pentamethyl-2-oxo-5-oxa-8-thiaspiro[3.4]oct-6-ene-7-carboxylate (3c): (a) Prepared using general procedure with thioketone **1a** (328 mg, 2.1 mmol) in diazoketoester **2c** (313 mg, 2.2 mmol), that was kept at room temperature for 60 days. The excess of diazo compound was removed in vacuo at 20 °C/0.1 mbar for 1 h and the obtained residue was recrystallized from Et_2O to give 1,3-oxathiole **3c** in 60% (77%) yield (341 mg, 1.26 mmol).

(b) Prepared as described above with thioketone **1a** (328 mg, 2.1 mmol) in diazoketoester **2c** (313 mg, 2.2 mmol), that was kept at 80 °C for 20 h, to furnish after the same workup procedure of the reaction mixture oxathiole **3c** in 64% (78%) yield (362 mg, 1.34 mmol).

3c: colourless solid. M.p. 83–85 °C. R_f = 0.46 (petroleum ether/diethyl ether, 2:1). ^{1}H NMR (600 MHz, $CDCl_3$): δ = 3.77 (s, 3 H, OC*H$_3$*), 2.29 (s, 3 H, C*H$_3$*C=O), 1.29, 1.23 (2 s, 12 H, 2(C*H$_3$*)$_2$C) ppm. ^{13}C NMR (151 MHz, $CDCl_3$): δ = 218.5 (*C2*=O), 163.6 (*C*=O ester), 159.4 (O-*C*=C), 102.8 (*C4*), 100.7 (O-C=*C*), 66.6 (2*C*Me$_2$), 56.0 (O*C*H$_3$), 22.9, 18.2 (4*C*H$_3$), 14.1 (*C*H$_3$C=C) ppm. IR (KBr): $\tilde{v}_{max}$ (I_{rel}) = 2982 (45), 1778 (85), 1713 (100), 1633 (100), 1281 (92), 1093 (97), 1058 (94), 786 (31), 759 (56) cm^{-1} (%). UV (EtOH): λ_{max} (lgε) = 205 (3.71), 274 (3.30), 307 (3.55) nm. HRMS (ESI): calcd. for $C_{13}H_{19}O_4S[M+H]^+$ 271.0999; found 271.0998.

Methyl 1,1,3,3-tetramethyl-2-oxo-6-(trifluoromethyl)-5-oxa-8-thiaspiro[3.4]oct-6-ene-7-carboxylate (3d): (a) The reaction was carried following the general procedure with thioketone **1a** (312 mg, 2.0 mmol) in diazoketoester **2d** (412 mg, 2.1 mmol) at room temperature for 60 d. After typical workup (see above) the obtained residue was subjected to ^{1}H NMR analysis, which allowed to determine the content of 1,3-oxathiole **3d**.

(b) In accordance with the general procedure using a solution of thioketone **1a** (156 mg, 1.0 mmol) in diazoketoester **2d** (206 mg, 1.05 mmol) at 80 °C for 72 h. After removal of the excess of diazo compound (20 °C/0.1 mbar, 1 h) and recrystallization of the residue from pentane, 1,3-oxathiole **3d** was obtained in 16% (24%) yield (52 mg, 0.16 mmol).

3d: colourless solid. M.p. 86–88 °C. R_f = 0.45 (petroleum ether/acetone, 5:1). ^{1}H NMR (600 MHz, $CDCl_3$): δ = 3.84 (s, 3 H, OC*H$_3$*), 1.31, 1.30 (2 s, 12 H, 2(C*H$_3$*)$_2$C) ppm. ^{13}C NMR (151 MHz, $CDCl_3$): δ = 216.7 (*C2*=O), 160.0 (*C*=O ester), 141.8 (q, *C6*, $J_{C,F}$ = 40.6 Hz), 117.7 (q, *CF$_3$*, $J_{C,F}$ = 273.3 Hz), 111.5 (q, *C7*, $J_{C,F}$ = 2.9 Hz), 105.0 (*C4*), 67.4 (2*C*Me$_2$), 53.1 (O*C*H$_3$), 22.7, 18.2 (4*C*H$_3$) ppm. IR (KBr): $\tilde{v}_{max}$ (I_{rel}) = 2977 (35), 1782 (68), 1740 (92), 1643 (77), 1331 (66), 1280 (68), 1151 (100), 1090 (67), 1039 (72), 795

(38), 758 (46), 722 (48) cm^{-1} (%). UV (EtOH): λ_{max} (lg ε) = 203 (3.77), 248 (2.90), 262 (3.05), 314 (3.41) nm. HRMS (ESI): calcd. for $C_{13}H_{15}NaF_3O_4S[M+Na]^+$ 347.0535; found 347.0542.

Methyl 6-methoxy-1,1,3,3-tetramethyl-2-oxo-5-oxa-8-thiaspiro[3.4]oct-6-ene-7-carboxylate (3e) and dimethyl 2-(2,2,4,4-tetramethyl-3-oxocyclobutylidene)propanedioate (5e): (a) The reaction was performed following the general procedure with a solution of thioketone **1a** (328 mg, 2.1 mmol) in diazomalonate **2e** (335 mg, 2.2 mmol) at room temperature for 65 days. After removal of the excess of diazo compound (20 °C/2 mbar, 1 h) and silica gel column chromatography (25 g silica gel, petroleum ether/acetone, from 30:1 to 10:1) the obtained fractions were recrystallized from petroleum ether to give oxathiole **3e** in 40% (65%) yield (238 mg, 0.83 mmol) and alkene **5e** in 13% (18%) yield (69 mg, 0.27 mmol).
(b) In accordance with the general procedure using a solution of thioketone **1a** (328 mg, 2.1 mmol) in diazomalonate **2e** (335 mg, 2.2 mmol) at 80 °C for 20 h. The residue after removal of the excess of diazo compound (60°C/2 mbar, 30 min) was recrystallized from pentane to produce alkene **5e** in 69% (81%) yield (371 mg, 1.46 mmol).
(c) 1,3-Oxathiole **3e** (57 mg, 0.2 mmol) was kept at room temperature in a tightly closed flask for 2 months, whereupon the sample of 1,3-oxathiole contained 7 mg (12%) of alkene **5e** (according to ^{1}H NMR spectral data). The ensuing heating of this sample at 80 °C for 2 h gave rise to disappearance of oxathiole **3e** and formation of a mixture of the alkene **5e** (25 mg, 49%) and oxathiole hydrolysis products [1].
3e [1]: colourless solid. M.p. 83–85°C. R_f = 0.39 (petroleum ether/ethyl acetate, 4:1). ^{1}H NMR (600 MHz, $CDCl_3$): δ = 4.02 (s, 3 H, OCH_3), 3.72 (s, 3 H, OCH_3 ester), 1.31, 1.30 (2 s, 12 H, $2(CH_3)_2C$) ppm. ^{13}C NMR (151 MHz, $CDCl_3$): δ = 217.5 (*C2*=O), 162.8 (*C*=O ester), 159.2 (O-*C*=C), 99.3 (*C4*), 75.8 (O-C=*C*), 66.6 (2*C*Me_2), 58.2 (O*C*H_3), 51.7 (O*C*H_3 ester), 22.5, 18.4 (4*C*H_3) ppm.
5e [1]: colourless solid. M.p. 62–63°C. R_f = 0.27 (petroleum ether/ethyl acetate, 4:1). ^{1}H NMR (600 MHz, $CDCl_3$): δ = 3.81 (s, 6 H, OCH_3 ester), 1.41 (s, 12 H, $2(CH_3)_2C$) ppm. ^{13}C NMR (151 MHz, $CDCl_3$): δ = 218.3 (*C*=O ketone), 171.2 (*C1'*), 164.4 (2 *C*=O ester), 122.2 (*C2*), 64.9 (2*C*Me_2), 52.3 (2O*C*H_3), 21.2 (4*C*H_3) ppm.

2',2',4',4'-Tetramethyl-4,5,6,7-tetrahydrospiro[1,3-benzoxathiole-2,1'-cyclobutane]-3',4-dione (3f): (a) The reaction was carried following the general procedure with a solution of thioketone **1a** (223 mg, 1.4 mmol) and diazodiketone **2f** (207 mg, 1.5 mmol)

in 1 mL of abs. toluene at 20 °C for 70 days. After removal of the solvent the obtained residue did not contain any new signals as determined by ^{1}H NMR spectroscopy.

(b) The reaction was carried out following the general procedure using a solution of thioketone **1a** (156 mg, 1.0 mmol) in diazodiketone **2f** (152 mg, 1.1 mmol) at 80 °C for 72 h. The residue after removal of the excess of diazo compound (80 °C/2 mbar, 30 min) and solvent was separated by chromatography (15 g silica gel, petroleum ether/acetone, from 20:1 to 5:1) to give oxathiole **3f** in 7% (14%) yield (19 mg, 0.07 mmol).

3f: colourless solid. R_f = 0.45 (petroleum ether/acetone, 5:1). ^{1}H NMR (600 MHz, $CDCl_3$): δ = 2.71-2.65, 2.43-2.39, 2.10-2.00 (3 m, 6 H, 3CH_2) 1.30, 1.24 (2 s, 12 H, 2(CH_3)$_2$C) ppm.

2',2',4',4',6,6-Hexamethyl-4,5,6,7-tetrahydrospiro[1,3-benzoxathiole-2,1'-cyclobutane]-3',4-dione (3g): (a) The reaction was carried out following the general procedure with a solution of thioketone **1a** (223 mg, 1.4 mmol) and diazodiketone **2g** (249 mg, 1.5 mmol) in 1 mL of abs. toluene at 20 °C for 70 days. After removal of the solvent the obtained residue did not contain any new signals as determined by ^{1}H NMR spectroscopy.

(b) The reaction was carried out following the general procedure using a solution of thioketone **1a** (156 mg, 1.0 mmol) in diazodiketone **2g** (183 mg, 1.1 mmol) at 80 °C for 90 h. The residue after removal of the excess of diazo compound (80 °C/2 mbar, 30 min) and solvent was separated by chromatography (15 g silica gel, petroleum ether/acetone, from 20:1 to 10:1) to give oxathiole **3g** in 18% (25%) yield (53 mg, 0.07 mmol).

3g: colourless solid. M.p. 150–152 °C (with decomposition). R_f = 0.40 (petroleum ether/acetone, 5:1).). ^{1}H NMR (300 MHz, $CDCl_3$): δ = 2.42, 2.33 (2 s, 4 H, 2CH_2), 1.31, 1.24, 1.13 (3 s, 18 H, 3(CH_3)$_2$C) ppm. ^{13}C NMR (75 MHz, $CDCl_3$): δ = 218.1 (*C3'*=O), 191.4 (*C4*=O), 166.6 (O-*C*=C), 110.3 (O-C=*C*), 106.8 (*C4*), 66.8 (2*C*Me$_2$), 51.0 (*C*H$_2$C=O), 38.3 (*C*H$_2$C=C), 28.5, 23.1, 18.2 (6*C*H$_3$) ppm. IR (KBr): $\tilde{\nu}_{max}$ (I_{rel}) = 2965 (41), 2934 (22), 2875 (15), 1797 (44), 1779 (58), 1649 (68), 1621 (100), 1457 (34), 1369 (40),1353 (48), 1244 (49), 1064 (60), 1041 (46), 1027 (42), 957 (23), 635 (7), 614 (10) cm^{-1} (%). UV (EtOH): λ_{max} (lg ε) = 207 (3.77), 278 (3.02), 325 (3.49) nm. HRMS (ESI): calcd. for $C_{16}H_{23}O_3S[M+H]^+$ 295.1362; found 295.1353.

3. Reactions of diazodicarbonyl compounds with adamanthanethione 1b:

4'-Acetyl-5'-methylspiro[adamantan-2,2'-[1,3]oxathiole] (7a): The reaction was performed according to the general procedure with diazodiketone **2a** (265 mg, 2.1 mmol). After removal of the excess of diazo compound and solvents at 50 °C/1 mbar for 2 h, separation of the residue by column chromatography (15 g silica gel, petroleum ether/acetone, from 30:1 to 25:1) followed by recrystallization from petroleum ether 1,3-oxathiole **7a** was obtained in 33% (48%) yield (174 mg, 0.66 mmol).
7a: colourless crystalls. M.p. 69–71 °C. R_f = 0.46 (petroleum ether/acetone, 5:1). ^{1}H NMR (300 MHz, $CDCl_3$): δ = 2.31 (br. s, 2 H, 2C*H*), 2.28, 2.25 (2 s, 6 H, 2CH_3), 2.20, 2.15 (2 br. s, 2 H, 2 C*H*), 1.84-1.61 (m, 10 H, 5 CH_2) ppm. ^{13}C NMR (151 MHz, $CDCl_3$): δ = 191.8 (*C*=O), 158.4 (O-*C*=C), 111.5 (O-C=*C*), 105.1 (C_q), 40.0 (2 *C*H), 37.2, 35.5, 33.2 (2:1:1, 4 *C*H_2), 30.3 (*C*H_3C=O), 26,4, 26,3 (2 *C*H), 15.8 (*C*H_3C=C) ppm. IR (KBr): $\tilde{\nu}_{max}$ (I_{rel}) = 2916 (58), 2886 (62), 1635 (68), 1594 (100), 1310 (60), 1248 (63), 1062 (62), 998 (58), 695 (31), 669 (30) cm^{-1} (%). UV (EtOH): λ_{max} (lgε) = 209 (3.39), 270 (2.47), 339 (3.15) nm. HRMS (ESI): calcd. for $C_{15}H_{21}O_2S[M+H]^+$ 265.1257; found 265.1264.

Methyl 5'-methylspiro[adamantan-2,2'-[1,3]oxathiole]-4'-carboxylate (7c): The reaction was performed according to the general procedure with diazoketoester **2c** (298 mg, 2.1 mmol). After removal of the excess of diazo compound and solvents at 50°C/1 mbar for 2 h and recrystallization of the residue from petroleum ether gave 1,3-oxathiole **7c** in 27% (39%) yield (153 mg, 0.55 mmol).
7c: colourless solid. M.p. 93–95 °C. R_f = 0.51 (petroleum ether/acetone, 5:1). ^{1}H NMR (300 MHz, $CDCl_3$): δ = 3.74 (s, 3 H, OCH_3), 2.32 (br. s, 2 H, 2C*H*), 2.27 (s, 3 H, CH_3), 2.20, 2.15 (2 br. s, 2 H, 2 C*H*), 1.85-1.61 (m, 10 H, 5 CH_2) ppm. ^{13}C NMR (151 MHz, $CDCl_3$): δ = 164.3 (*C*=O), 159.7 (O-*C*=C), 105.3 (C_q), 100.1 (O-C=*C*), 53.6 (*C*H_3C=O), 39.9 (2 *C*H), 37.2, 35.5, 33.2 (2:1:1, 4 *C*H_2), 26.4, 26.3 (2 *C*H), 15.0 (*C*H_3C=C) ppm. IR (KBr): $\tilde{\nu}_{max}$ (I_{rel}) = 2921 (58), 2896 (60), 2856 (50), 1704 (100), 1622 (85), 1437 (55), 1369 (53), 1275 (86), 1261 (88), 1090 (91), 989 (62), 786 (28), 758 (48), 692 (23) cm^{-1} (%). UV (EtOH): λ_{max} (lgε) = 267 (3.10), 313 (3.52) nm. HRMS (ESI): calcd. for $C_{15}H_{21}O_3S[M+H]^+$ 281.1206; found 281.1218.
Dimethyl spiro[adamantan-2,2'-thiirane]-3',3'-dicarboxylate (8e): obtained using general procedure with diazomalonate **2e** (332 mg, 2.1 mmol). After removal of excess diazo compound and solvents at 20 °C/1 mbar for 5 h and column chromatography (15 g silica gel, petroleum ether/acetone, from 30:1 to 10:1) thiirane **8e** was isolated in 7% (13%) yield (43 mg, 0.16 mmol).

8e: colourless solid. M.p. 171–173 °C. R_f = 0.40 (petroleum ether/acetone, 5:1). ^1H NMR (300 MHz, $CDCl_3$): δ = 3.76 (s, 6 H, 2OC*H*3), 3,18, 2.62, 258 (3 br. s, 2:1:1, 4 H, 4 C*H*), 2.19-1.88 (m, 10 H, 5 C*H*2) ppm. ^{13}C NMR (151 MHz, $CDCl_3$): δ = 169.1 (2 *C*=O), 65.6 ($C^{3'}$), 53.2 ($C^{2'}$), 39.2 (2 *C*H), 38.1, 37.4, 35.5 (2:1:1, 4 CH_2), 27.3, 26.3 (2 *C*H) ppm. HRMS (ESI): calcd. for $C_{15}H_{21}O_4S[M+H]^+$ 297.1155; found 297.1149.

References

[1] Mlostoń, G.; Romański, J.; Heimgartner, H. *Pol. J. Chem.* **2002**, *76*, 551–555.

7-Acetyl-1,1,3,3,6-pentamethyl-5-oxa-8-thiaspiro[3.4]oct-6-ene-2-one (**3a**):

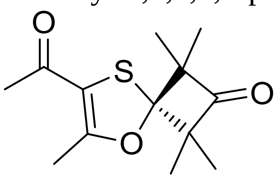

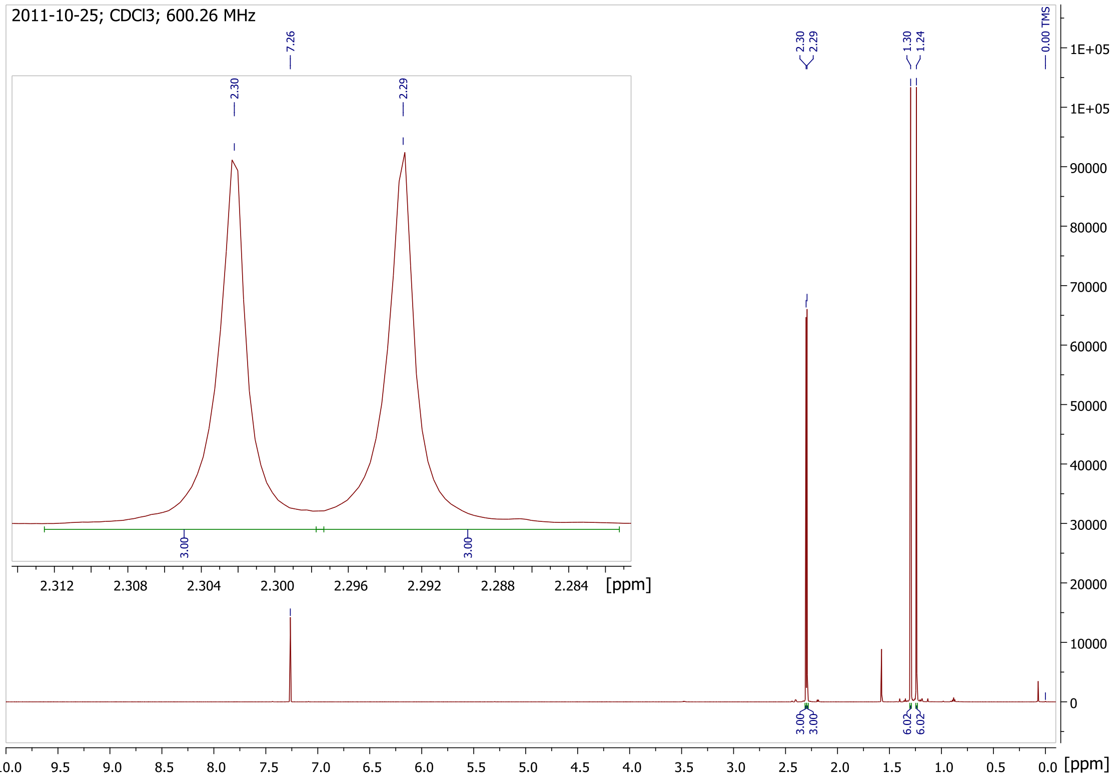

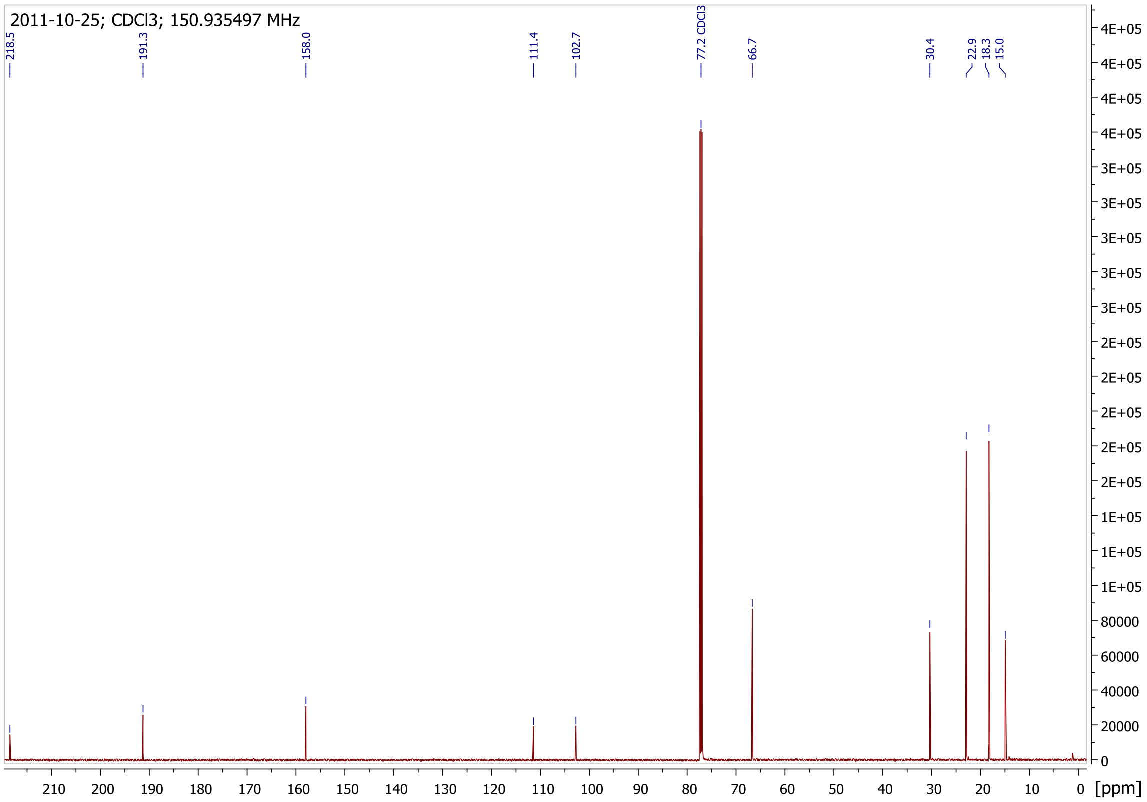

7-Benzoyl-1,1,3,3,-tetramethyl-6-phenyl-5-oxa-8-thiaspiro[3.4]oct-6-ene-2-one (**3b**):

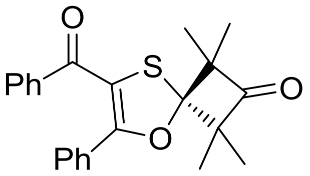
O
Ph
S
O
O
Ph

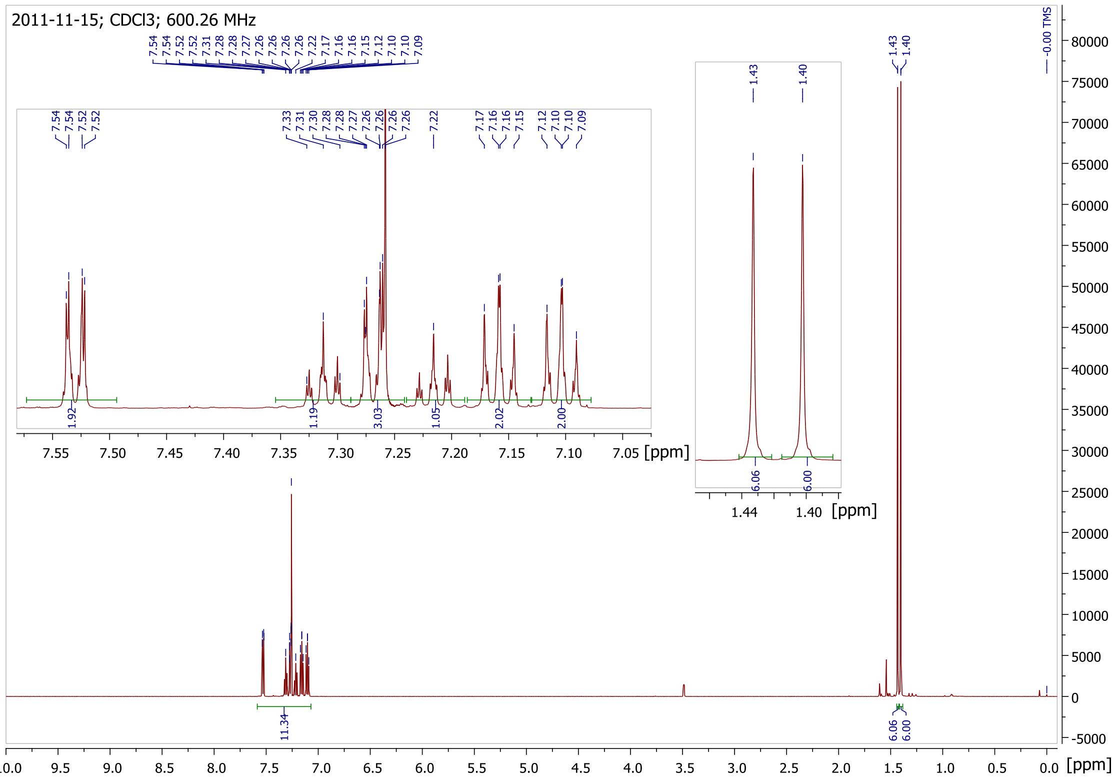
2011-11-15; CDCl3; 600.26 MHz
7.54 7.54 7.52 7.52 7.31 7.28 7.28 7.27 7.26 7.26 7.26 7.26 7.22 7.17 7.16 7.16 7.15 7.12 7.10 7.10 7.09
7.33 7.31 7.30 7.28 7.28 7.27 7.26 7.26 7.26
1.43 1.40
-0.00 TMS
1.92
1.19
3.03
1.05
2.02
2.00
6.06
6.00
11.34
[ppm]

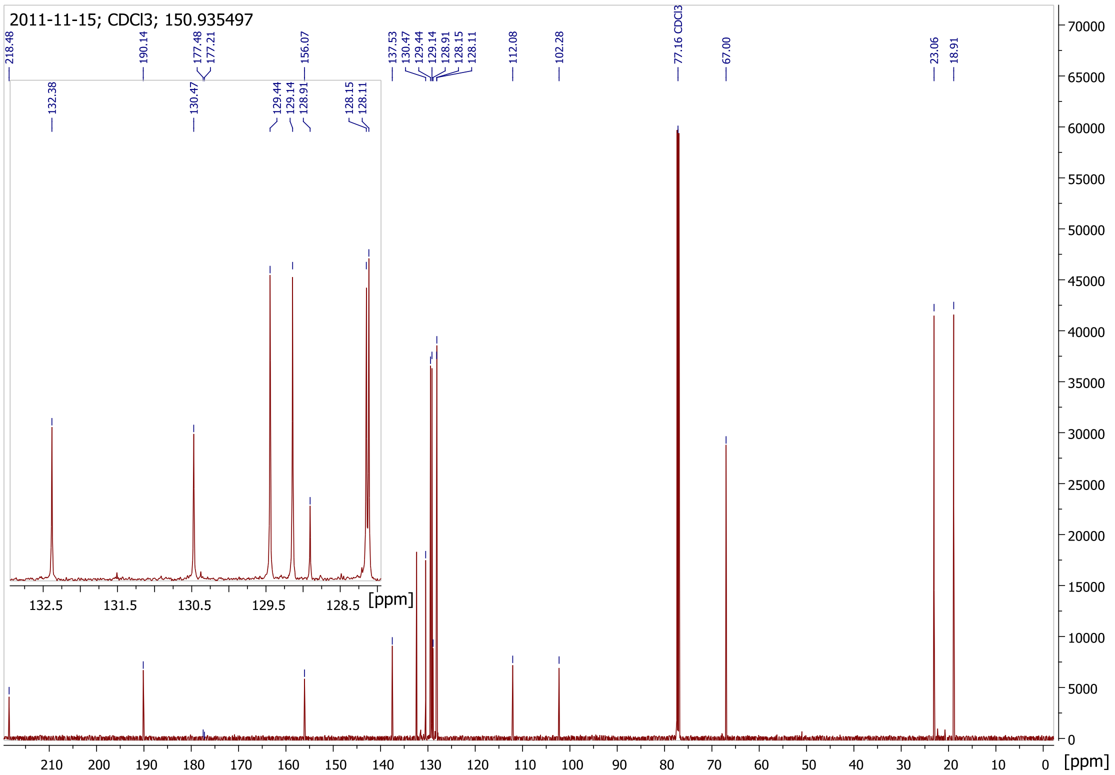
2011-11-15; CDCl3; 150.935497
218.48
190.14
177.48 177.21
156.07
137.53 130.47 129.44 129.14 128.91 128.15 128.11
132.38
112.08
102.28
77.16 CDCl3
67.00
23.06 18.91
[ppm]

Methyl 1,1,3,3,6-pentamethyl-2-oxo-5-oxa-8-thiaspiro[3.4]oct-6-ene-7-carboxylate (**3c**):

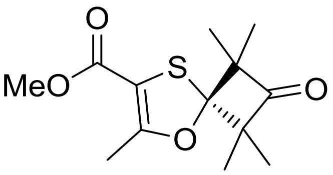

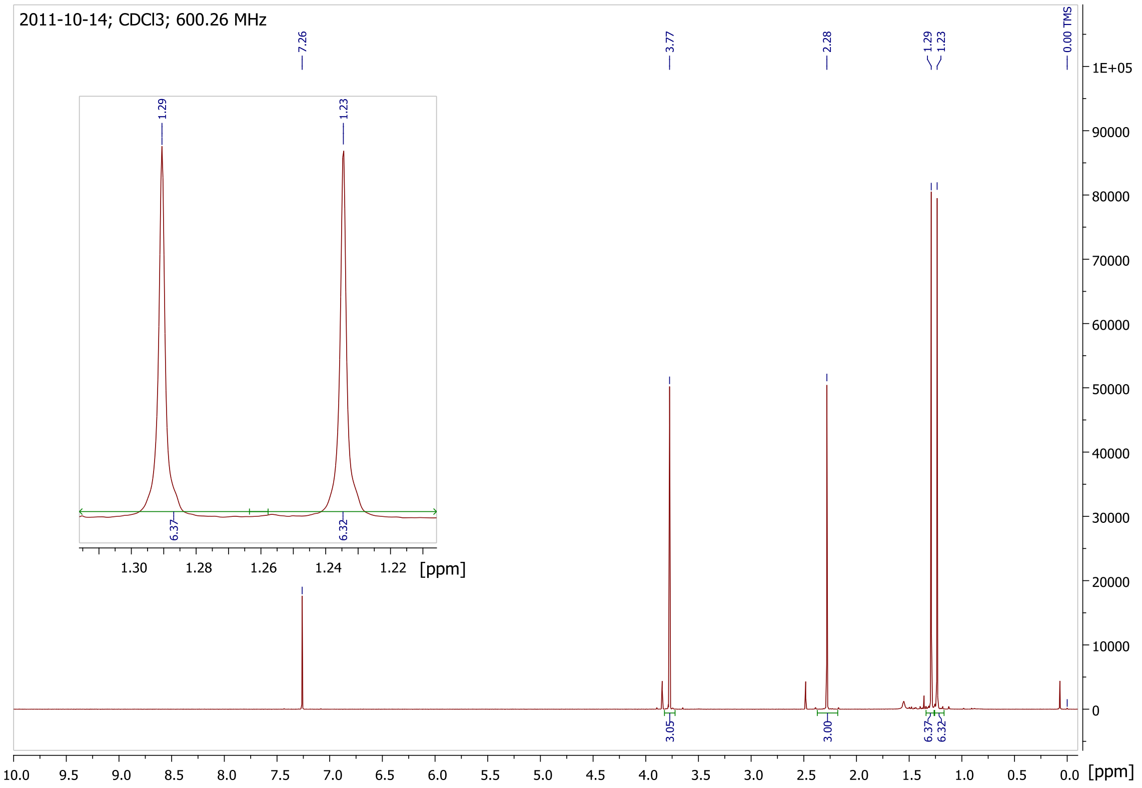

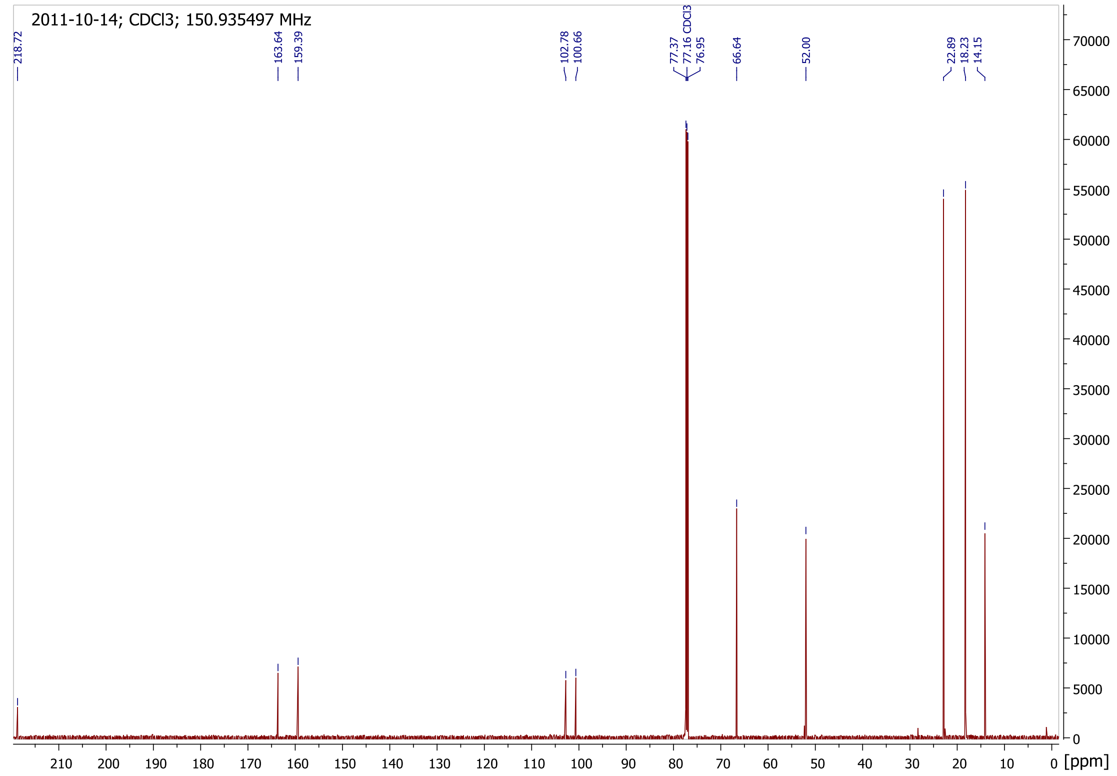

Methyl 1,1,3,3-tetramethyl-2-oxo-6-(trifluoromethyl)-5-oxa-8-thiaspiro[3.4]oct-6-ene-7-carboxylate (**3d**):

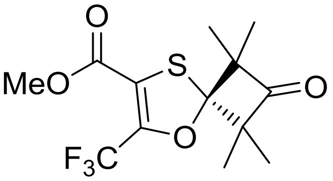

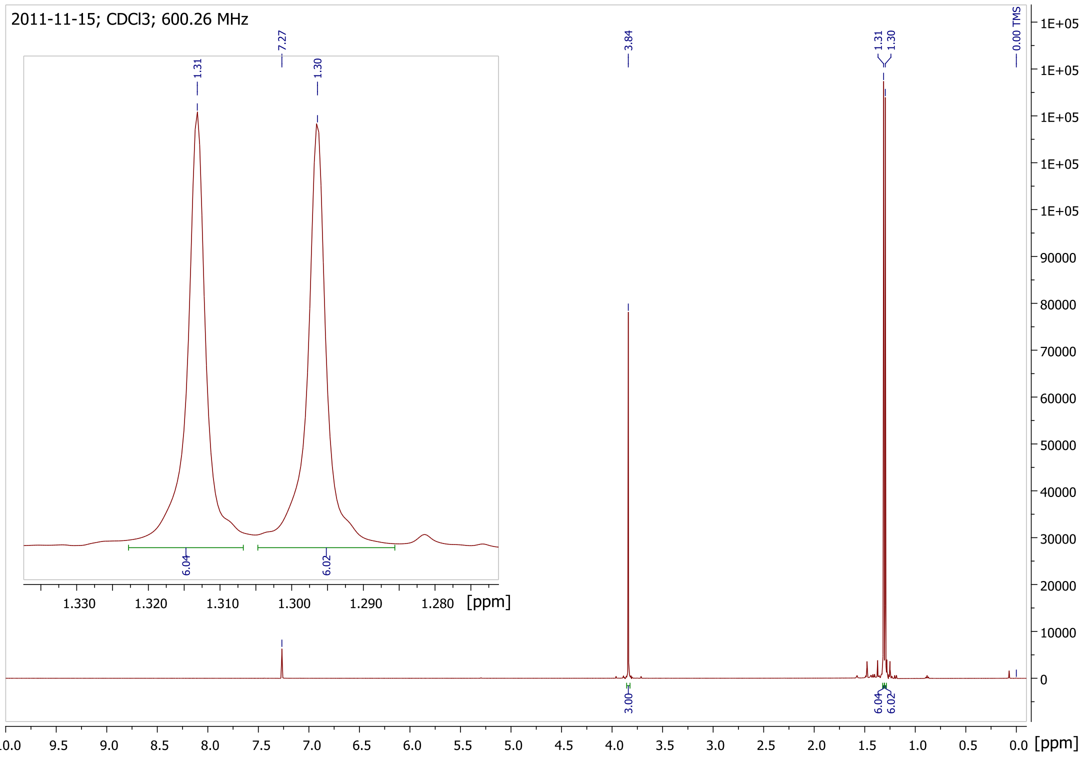

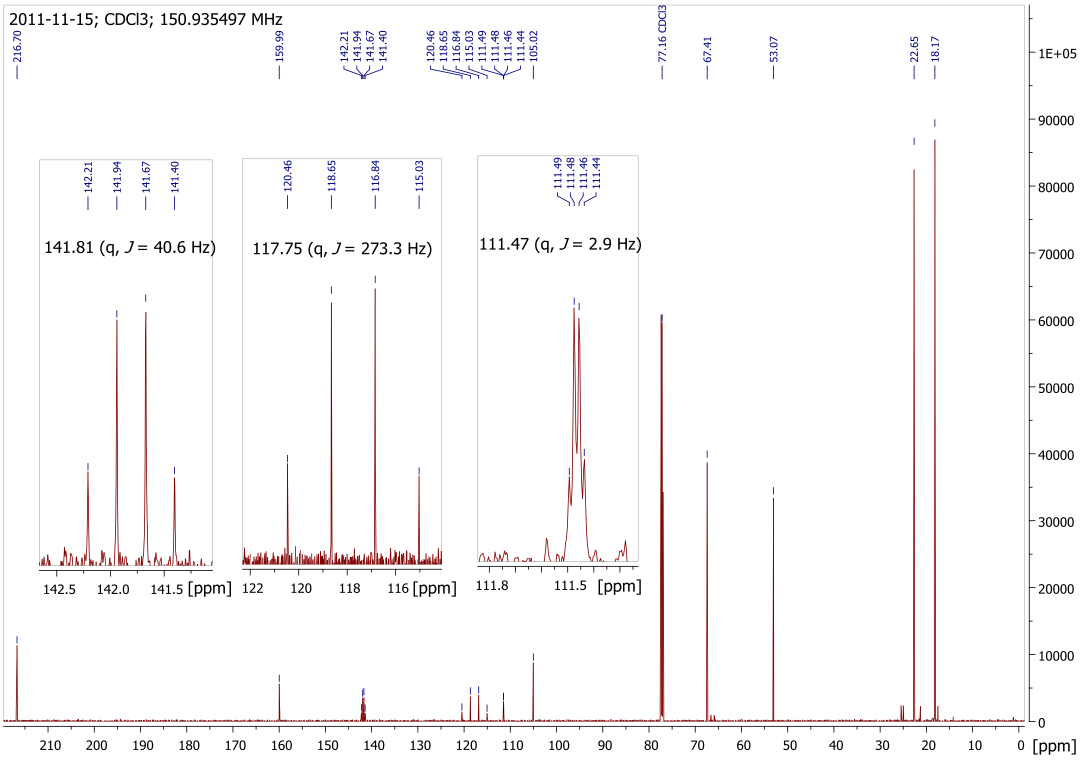

2',2',4',4'-Tetramethyl-4,5,6,7-tetrahydrospiro[1,3-benzoxathiole-2,1'-cyclobutane]-3',4-dione **(3f)**:

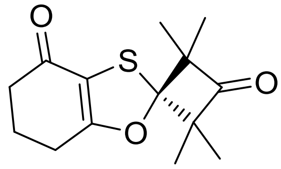

The compound **3f** wasn't obtained in pure form.

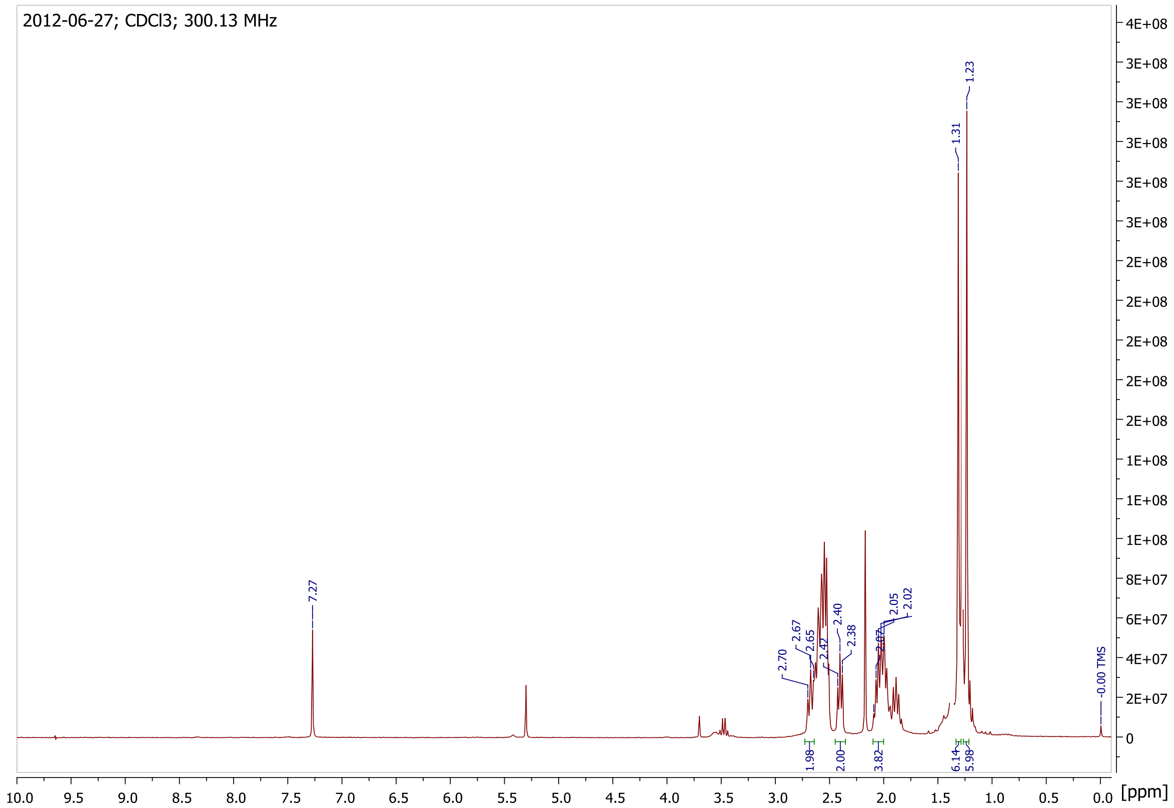

2',2',4',4',6,6-Hexamethyl-4,5,6,7-tetrahydrospiro[1,3-benzoxathiole-2,1'-cyclobutane]-3',4-dione (**3g**):

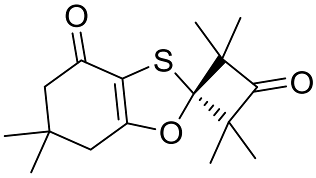

The compound **3f** wasn't obtained in pure form.

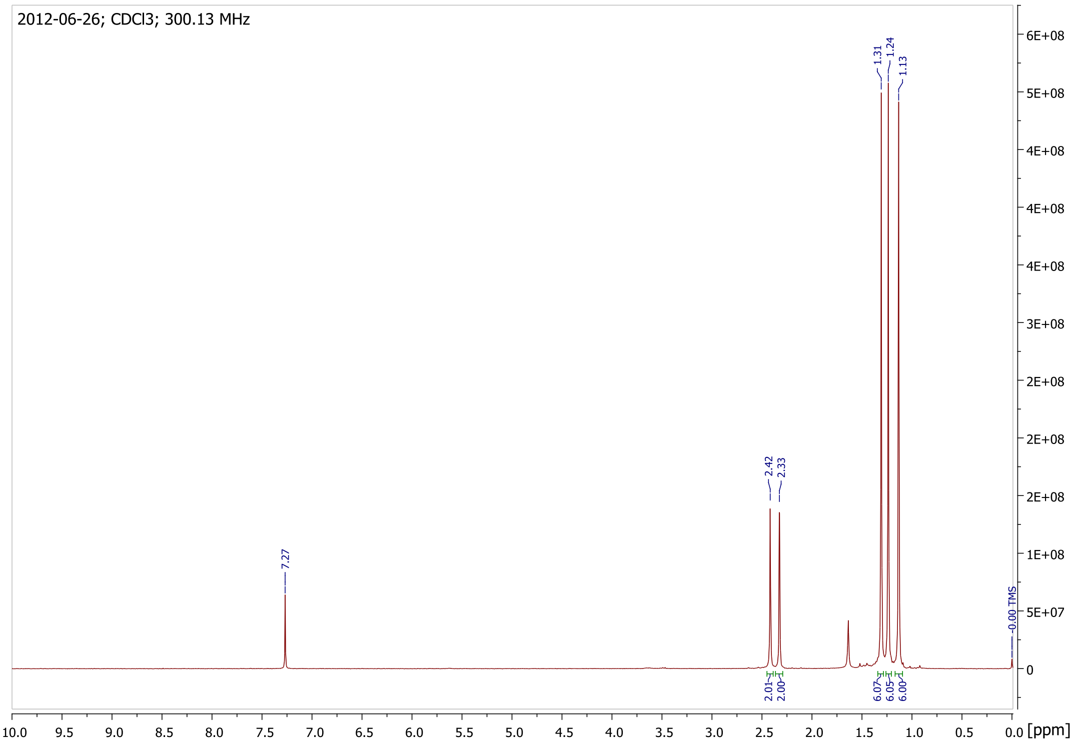

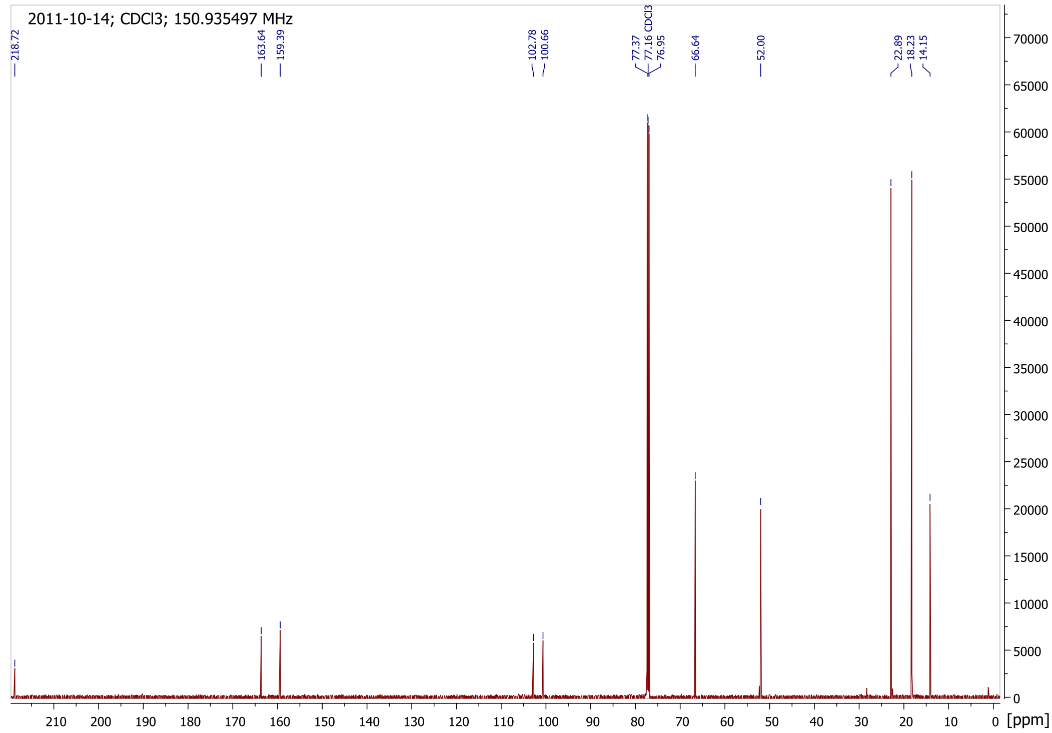

4'-Acetyl-5'-methylspiro[adamantan-2,2'-[1,3]oxathiole] (**7a**):

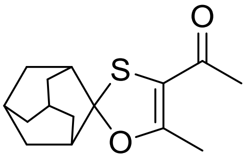

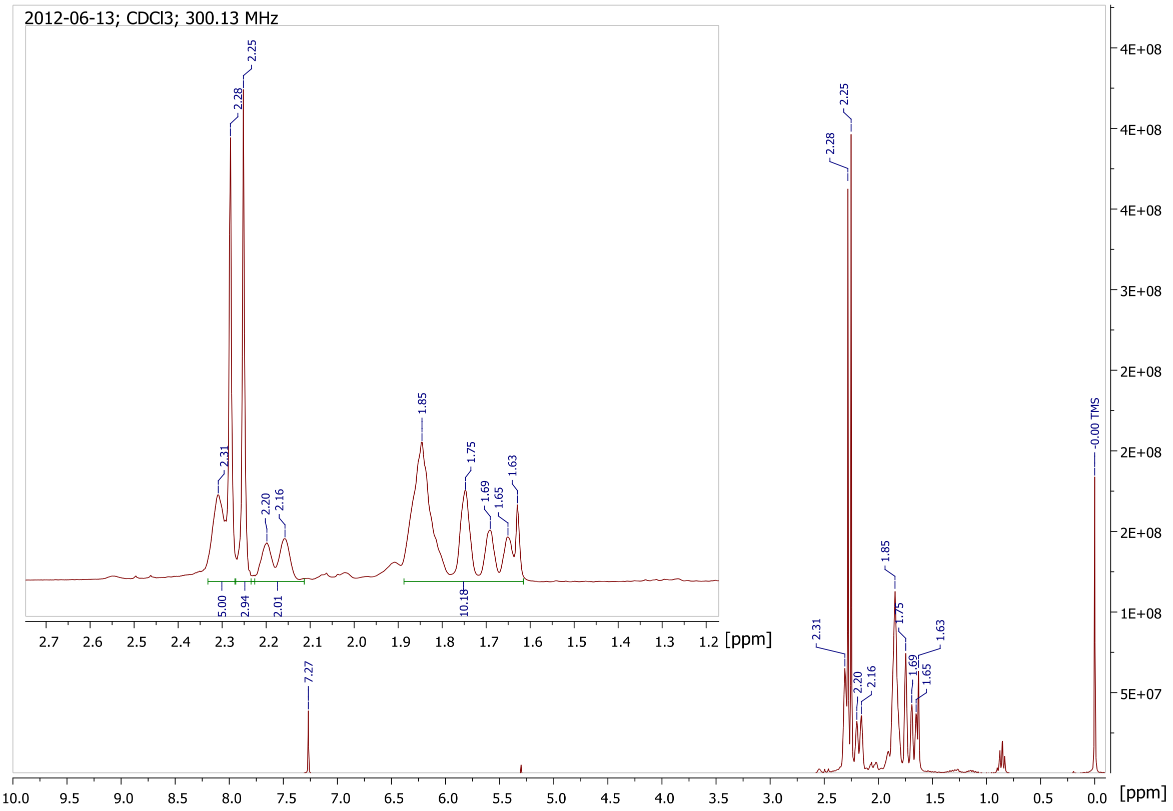

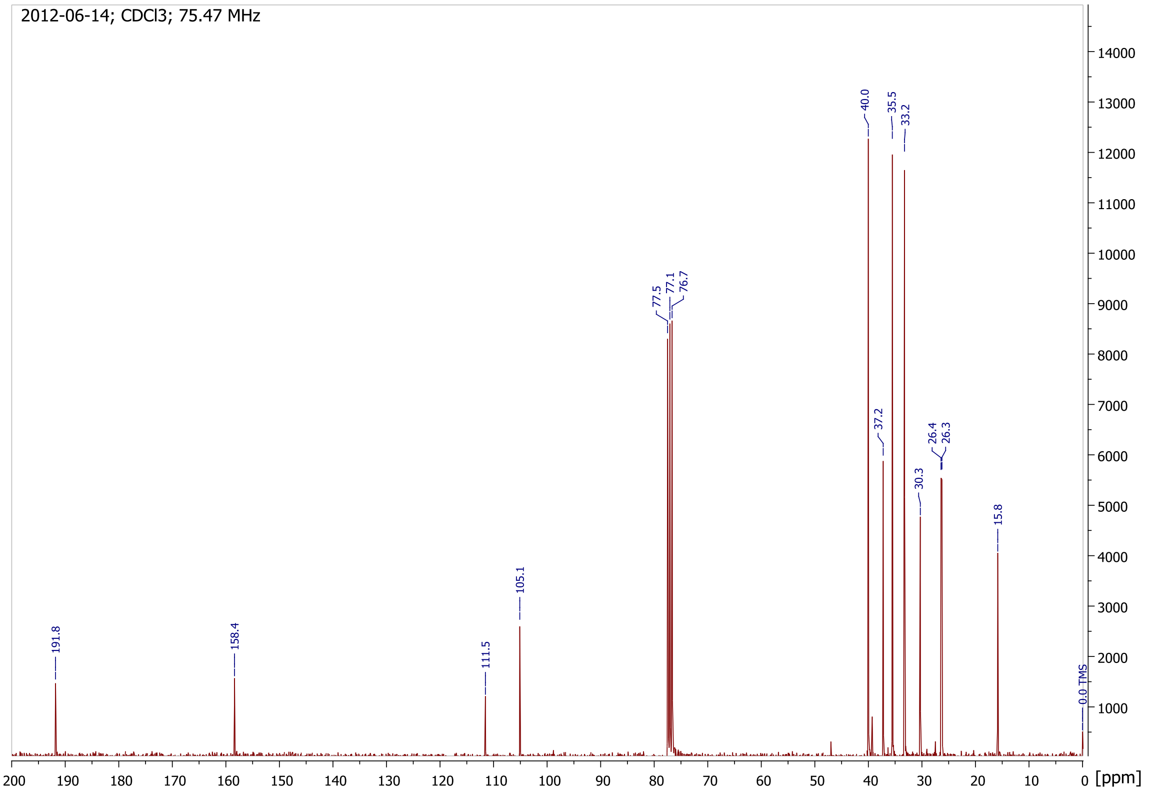

Methyl 5'-methylspiro[adamantan-2,2'-[1,3]oxathiole]-4'-carboxylate (**7c**):

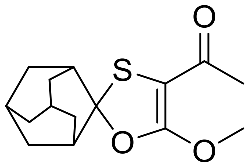

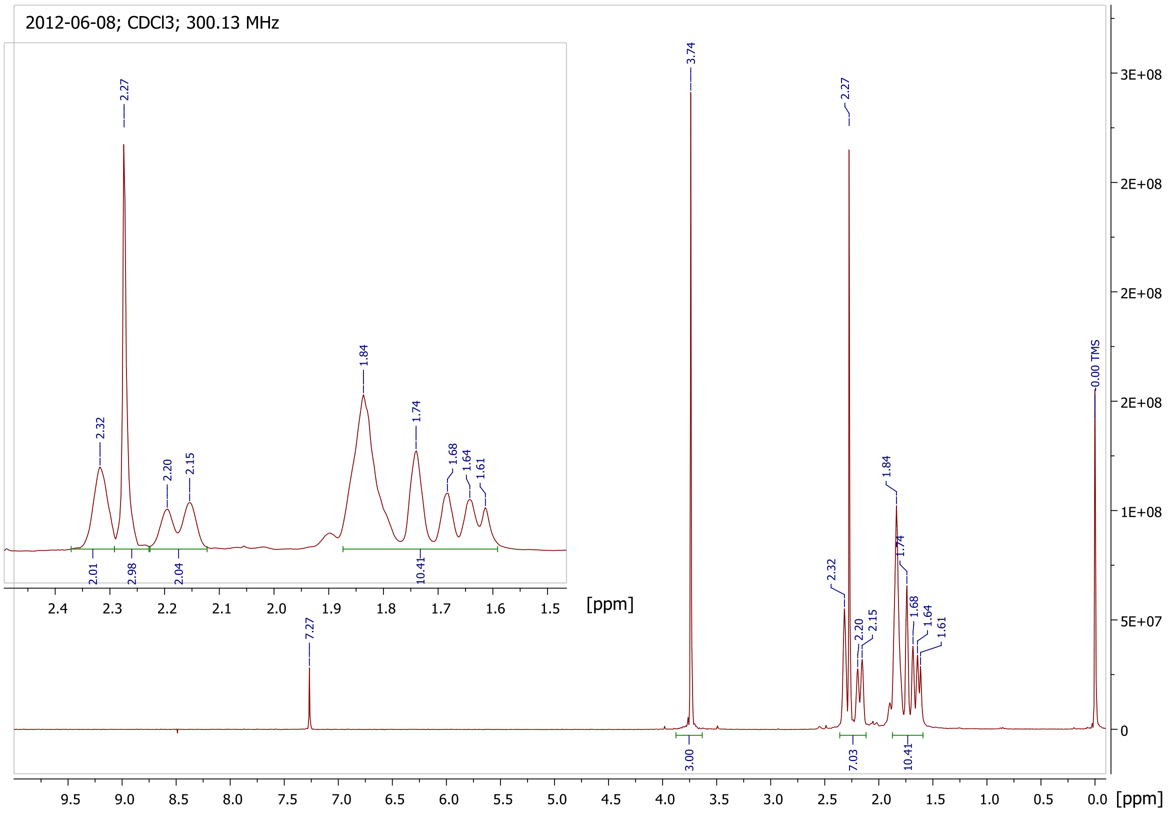

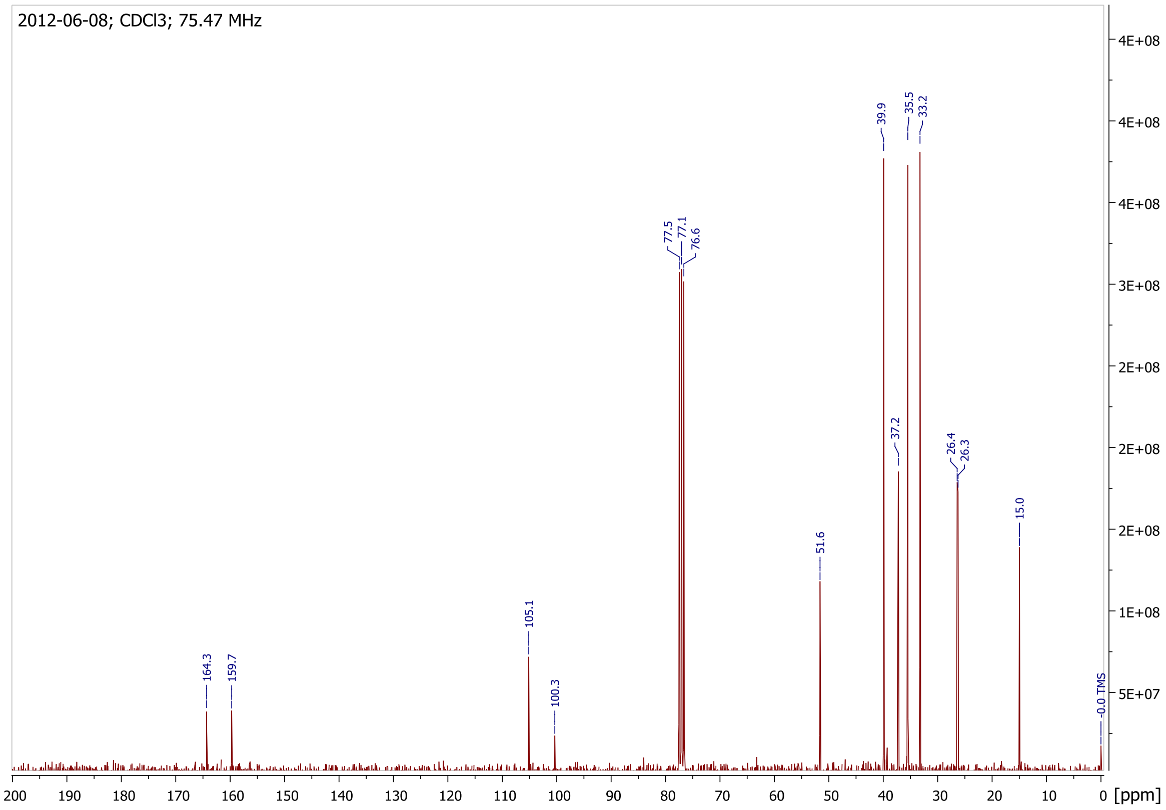

Dimethyl spiro[adamantan-2,2'-thiirane]-3',3'-dicarboxylate (**8e**):

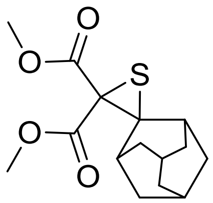

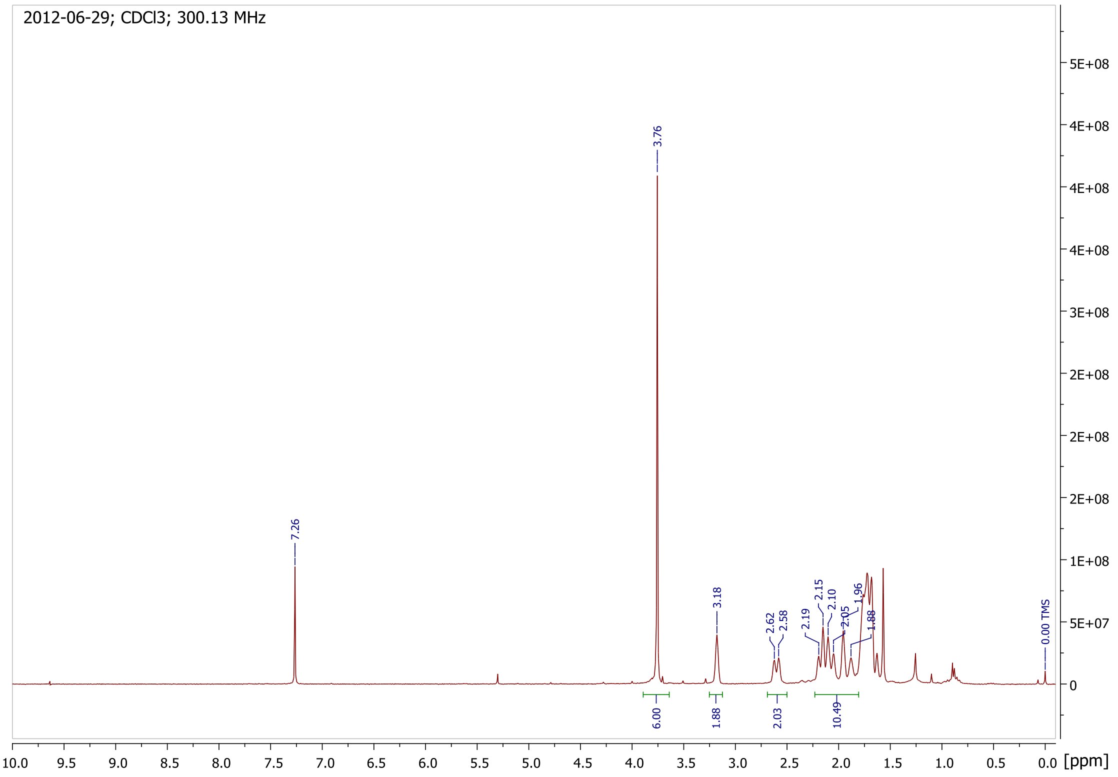

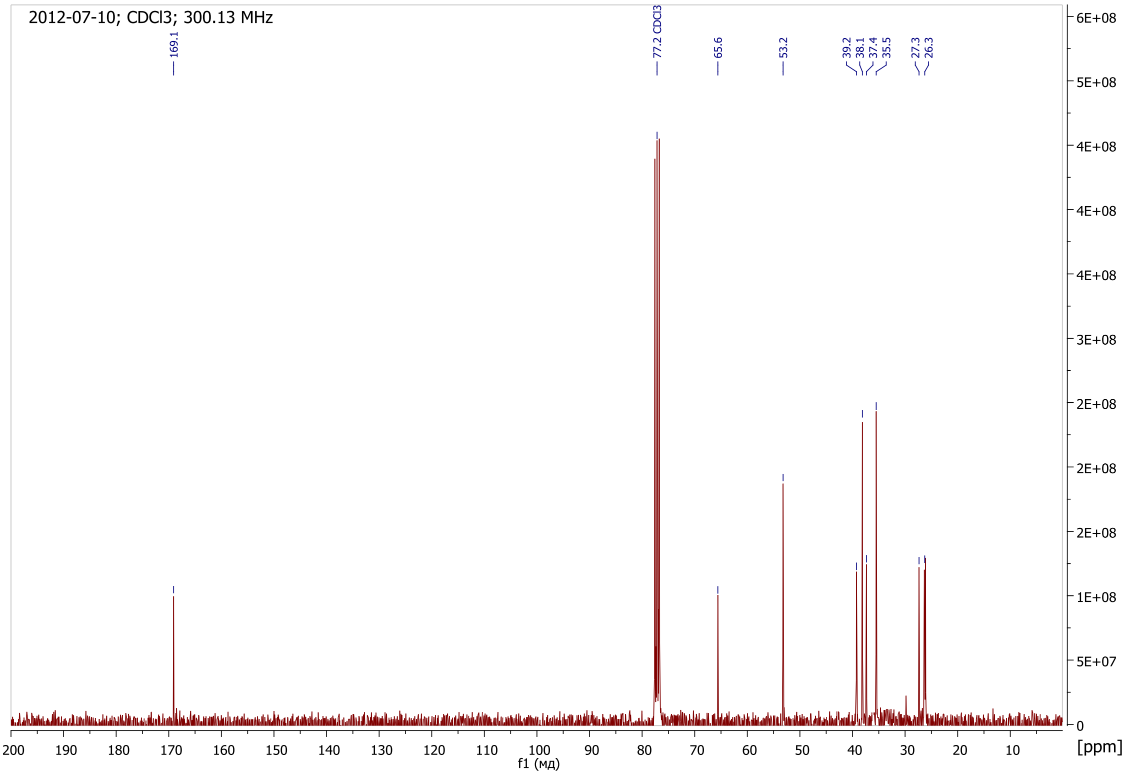

Dimethyl 3,3-diphenyl-2,2-dicarboxylate:

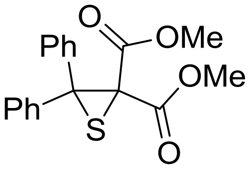

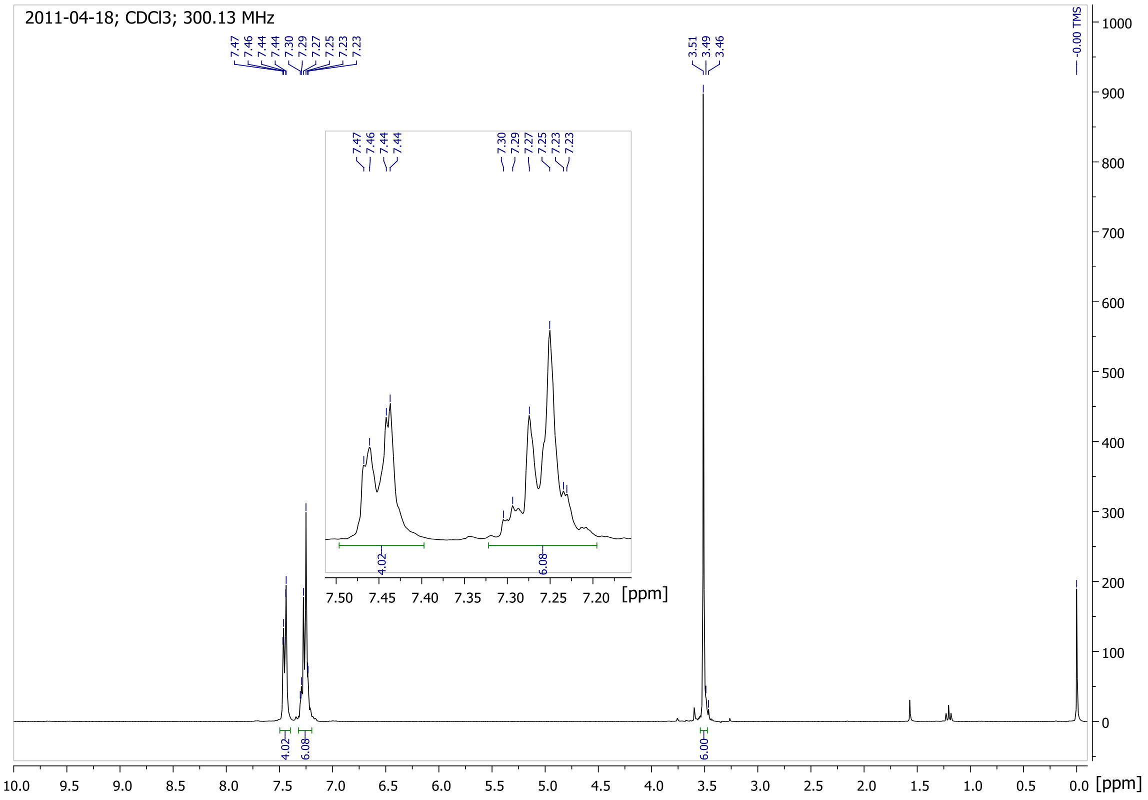

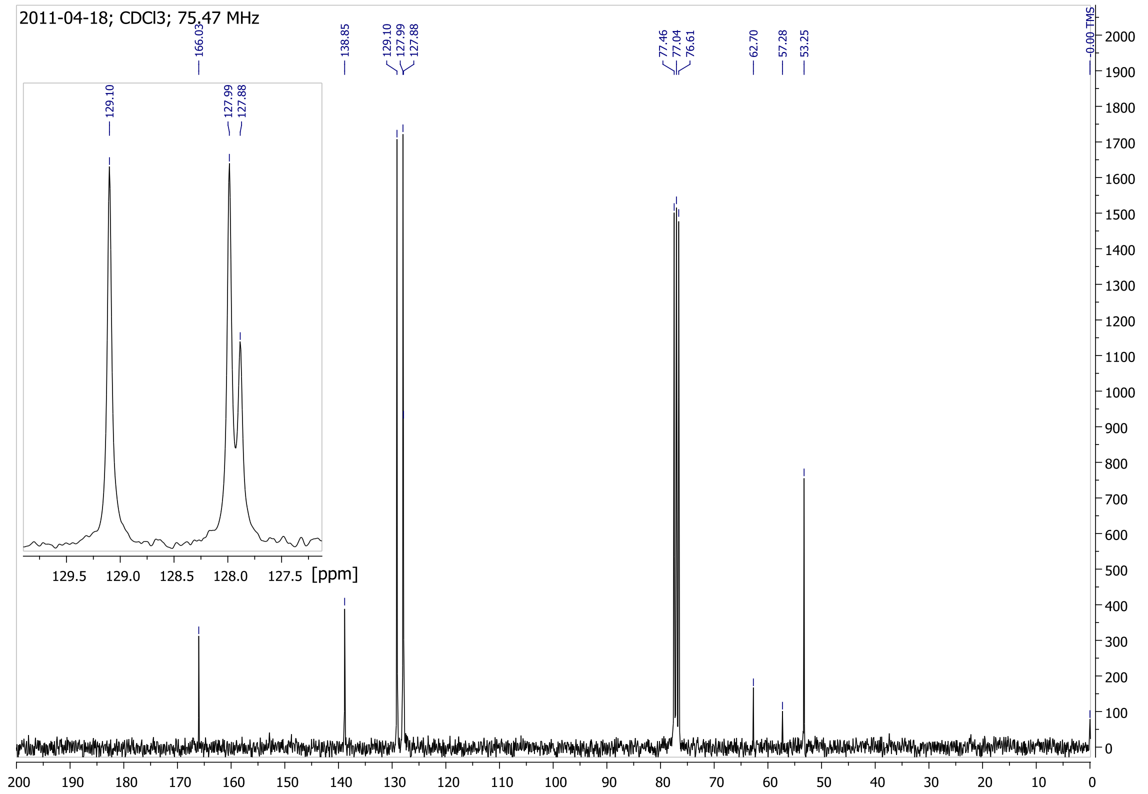

Supplement: File 1 — Experimental details for the preparation of the compounds 3a–g, 5e, 7a,c, 8e, their spectroscopic and analytical data, and 1H and 13C NMR spectra. [file Beilstein_J_Org_Chem-09-2751-s001.pdf]
