# Supplementary material for: Less reactive dipoles of diazodicarbonyl compounds in reaction with cycloaliphatic thioketones – First evidence for the 1,3-oxathiole–thiocarbonyl ylide interconversion
Source: Beilstein J Org Chem. 2013 Dec 2;9:2751–61. doi: 10.3762/bjoc.9.309 (PMC3869254; doi:10.3762/bjoc.9.309)
Supplement: File 2 — Details of computational studies: Cartesian coordinates, computed geometries of compounds, transition states, and computed total energies. [file Beilstein_J_Org_Chem-09-2751-s002.pdf]

Supporting Information

for

Less reactive dipoles of diazodicarbonyl compounds in reaction with cycloaliphatic thioketones – First evidence for the 1,3-oxathiole–thiocarbonyl ylide interconversion

Valerij A. Nikolaev*[1], Alexey V. Ivanov[1], Ludmila L. Rodina[1] and Grzegorz Mlostoń*[2]

Address: [1]Saint-Petersburg State University, 198504, Saint Petersburg, University prosp., 26, Russia and [2]University of Łódź, Faculty of Chemistry, Tamka 12, 91-403, Łódź, Poland

E-mail: Grzegorz Mlostoń - gmloston@uni.lodz.pl and Valerij A. Nikolaev - vnikola@VN6646.spb.edu

*Corresponding author

Details of computational studies: Cartesian coordinates, computed geometries of compounds, transition states and computed total energies.

Table S1: Details of computational results: Cartesian coordinates and computed total energies computed at the DFT PBE1PBE/6-31G(d) level.

Diazoacetylacetone **2a**

C	0.000008	0.314543	2.549146
H	-0.884893	0.961704	2.572851
H	0.884911	0.961703	2.572826
H	0.000020	-0.324177	3.433379
C	-0.000011	-0.574656	1.323749
C	-0.000007	0.107378	0.000000
C	-0.000011	-0.574656	-1.323749
C	0.000008	0.314543	-2.549146
H	0.884911	0.961703	-2.572826
H	0.000020	-0.324177	-3.433379
H	-0.884893	0.961704	-2.572851
O	-0.000011	-1.781228	1.402327
O	-0.000011	-1.781228	-1.402327
N	0.000007	1.417212	0.000000
N	0.000019	2.551113	0.000000

-453.455350 A.U.

2,2,4,4-tetramethyl-3-thioxocyclobutane-1-one **1a**

C	0.000005	0.748165	-0.000099
C	-1.119758	-0.292112	-0.000012
C	1.119754	-0.292126	-0.000019
C	-0.000009	-1.351126	-0.000004
C	-1.980834	-0.285391	1.262782
H	-1.373159	-0.301141	2.173244
H	-2.599351	0.618001	1.281175
H	-2.632408	-1.165199	1.270007
C	-1.980961	-0.285444	-1.262717
H	-1.373380	-0.301233	-2.173241
H	-2.632533	-1.165255	-1.269832
H	-2.599486	0.617943	-1.281092
C	1.980888	-0.285408	1.262731
H	2.632450	-1.165226	1.269935
H	2.599423	0.617974	1.281088
H	1.373256	-0.301139	2.173221

C	1.980899	-0.285477	-1.262768
H	2.599428	0.617907	-1.281179
H	2.632463	-1.165293	-1.269905
H	1.373276	-0.301270	-2.173263
O	-0.000017	-2.551544	-0.000291
S	0.000015	2.361237	0.000176

-784.702888 A.U.

thiadiazoline **10a**

S	0.461915	-0.336222	1.191451
C	1.686044	-0.107036	-0.145589
C	2.858927	-1.083578	-0.011502
O	3.252029	-1.397839	1.088993
C	3.489151	-1.567713	-1.286419
H	2.768343	-2.171010	-1.849806
H	3.752340	-0.727302	-1.938358
H	4.373180	-2.161343	-1.047848
C	2.151352	1.380586	-0.064134
O	1.612123	2.213861	-0.751616
C	3.233719	1.701683	0.928218
H	3.105922	1.149222	1.862753
H	4.206055	1.407035	0.513413
H	3.240087	2.777771	1.109346
C	-0.870457	-0.178672	-0.052227
C	-1.829601	1.090814	-0.066110
C	-2.114770	-1.135631	0.117926
C	-2.996442	0.101716	-0.066569
C	-1.767416	1.943247	1.197210
H	-1.805953	1.349830	2.115712
H	-0.841103	2.526820	1.210373
H	-2.617288	2.633646	1.201382
C	-1.773485	1.974020	-1.310118
H	-1.890349	1.395057	-2.229593
H	-2.585733	2.706437	-1.259604
H	-0.816580	2.502421	-1.358327
C	-2.269642	-1.722154	1.520898
H	-3.269068	-2.159491	1.613717

H	-1.525726	-2.506906	1.692461
H	-2.160650	-0.971547	2.311024
C	-2.290985	-2.220448	-0.936781
H	-1.535027	-3.004065	-0.810374
H	-3.280215	-2.675022	-0.820453
H	-2.208118	-1.824014	-1.951149
O	-4.182161	0.240571	-0.203135
N	0.984402	-0.293195	-1.418662
N	-0.240840	-0.332767	-1.360002

-1238.165227 A.U.

TS for formation of thiadiazoline **10a** from reagents

S	-0.305013	0.000002	1.366696
C	-2.071441	0.000001	-0.286401
C	-2.762069	1.318326	-0.000984
O	-3.577422	1.387759	0.883399
C	-2.351794	2.499376	-0.843177
H	-2.516044	2.305818	-1.909131
H	-2.937426	3.365216	-0.531284
H	-1.286523	2.714198	-0.703309
C	-2.762070	-1.318324	-0.000982
O	-3.577415	-1.387758	0.883408
C	-2.351807	-2.499371	-0.843185
H	-2.516067	-2.305808	-1.909137
H	-1.286535	-2.714197	-0.703329
H	-2.937439	-3.365211	-0.531289
C	0.994055	0.000000	0.323643
C	2.038325	-1.128030	0.087963
C	2.038328	1.128028	0.087961
C	3.013405	-0.000003	-0.279716
C	2.425604	-1.842265	1.387324
H	2.651368	-1.141215	2.196015
H	1.596079	-2.476370	1.718150
H	3.307151	-2.469909	1.218694
C	1.824684	-2.156766	-1.016205
H	1.572575	-1.698553	-1.975620
H	2.746282	-2.732493	-1.152359

H	1.028691	-2.857764	-0.738370
C	2.425608	1.842265	1.387320
H	3.307157	2.469906	1.218689
H	1.596085	2.476373	1.718145
H	2.651370	1.141216	2.196013
C	1.824689	2.156762	-1.016209
H	1.028699	2.857763	-0.738377
H	2.746290	2.732486	-1.152365
H	1.572579	1.698547	-1.975624
O	4.121245	-0.000004	-0.746096
N	-1.191809	0.000000	-1.288925
N	-0.077970	0.000000	-1.597138

-1238.110114 A.U.

TS for decomposition of thiadiazoline **10a** to dinitrogen and thiocarbonyl ylide **6a**

S	-0.420529	-0.912346	-0.869430
C	-1.845154	-0.118897	-0.270216
C	-2.965461	-1.099196	-0.215136
O	-2.907872	-2.126447	-0.871879
C	-4.137928	-0.866897	0.712191
H	-3.826046	-0.462120	1.679200
H	-4.863624	-0.175764	0.271498
H	-4.630973	-1.830950	0.853289
C	-1.980982	1.342004	-0.351037
O	-1.115576	2.025203	-0.885433
C	-3.183335	2.028801	0.256856
H	-4.089443	1.819672	-0.321958
H	-3.362049	1.713739	1.288710
H	-2.994932	3.103543	0.228996
C	0.900704	-0.214664	0.004937
C	1.743853	1.093313	-0.154359
C	2.178927	-1.093581	0.119733
C	2.962085	0.222373	0.173028
C	1.814287	1.556362	-1.618429
H	2.015166	0.733908	-2.310632
H	0.865410	2.018517	-1.892805
H	2.627318	2.283910	-1.711000

C	1.531340	2.281049	0.772107
H	1.526561	1.987133	1.825381
H	2.359859	2.982140	0.628522
H	0.593921	2.787221	0.527845
C	2.499695	-1.945386	-1.114105
H	3.527388	-2.312715	-1.028722
H	1.827874	-2.808830	-1.165236
H	2.413521	-1.387943	-2.050468
C	2.294359	-1.953897	1.377092
H	1.535371	-2.743988	1.364613
H	3.283330	-2.422261	1.397918
H	2.174541	-1.368439	2.291326
O	4.106376	0.480947	0.428456
N	-0.977210	0.075905	1.814583
N	0.149713	-0.039498	1.686742

-1238.123840 A.U.

Thiocarbonyl ylide **6a**

S	-0.418096	-1.057951	-0.536825
C	-1.864912	-0.169316	-0.158028
C	-2.801660	-1.124724	0.389091
O	-2.396057	-2.258799	0.677077
C	-4.262017	-0.802471	0.615768
H	-4.388680	0.022402	1.324166
H	-4.769459	-0.523096	-0.312631
H	-4.732079	-1.698109	1.025764
C	-1.991859	1.178380	-0.664370
O	-1.054117	1.766097	-1.208788
C	-3.320646	1.903863	-0.547712
H	-4.099133	1.422537	-1.148381
H	-3.678174	1.947448	0.485474
H	-3.167565	2.918497	-0.919839
C	0.934947	-0.218430	-0.202421
C	1.390579	0.910971	0.712313
C	2.341503	-0.736403	-0.494122
C	2.783774	0.260741	0.592988
C	1.429043	2.337930	0.146364

H	1.803597	2.367332	-0.879573
H	0.432212	2.777924	0.141192
H	2.100429	2.925433	0.781510
C	0.769842	0.889245	2.107313
H	0.782709	-0.113679	2.544921
H	1.337617	1.559167	2.760804
H	-0.267555	1.233572	2.064312
C	2.859735	-0.375936	-1.892765
H	3.942937	-0.531207	-1.927504
H	2.386340	-1.022315	-2.639294
H	2.649572	0.662915	-2.160691
C	2.615692	-2.201224	-0.165067
H	2.145989	-2.858901	-0.904290
H	3.695846	-2.377825	-0.180150
H	2.236512	-2.471049	0.825170
O	3.821961	0.439005	1.165726

-1128.772659 A.U.

N_2 molecule

N	0.000000	0.000000	0.551300
N	0.000000	0.000000	-0.551300

-109.413037 A.U.

Oxathiole **3e**

S	-0.216169	-1.537250	-0.368578
C	-1.523256	-0.357050	-0.155893
C	-2.882843	-0.841513	-0.025753
O	-3.174004	-2.021947	-0.040859
C	-5.131875	-0.303262	0.229331
H	-5.735306	0.599595	0.329897
H	-5.432286	-0.866699	-0.658519
H	-5.253318	-0.944071	1.107217
O	-3.787247	0.146989	0.112443
C	-1.038254	0.903867	-0.260390
O	0.292537	1.035554	-0.409204
O	-1.740692	2.021476	-0.282282
C	-1.039179	3.259889	-0.211017
H	-1.815218	4.024827	-0.231761
H	-0.467619	3.334661	0.718811
H	-0.369482	3.383615	-1.066115
C	0.997715	-0.187742	-0.137771
C	1.803112	-0.087173	1.219168
C	2.328580	-0.252434	-0.960513
C	3.063649	0.028826	0.354837

C	1.526914	1.136168	2.083491
H	1.576010	2.065510	1.509824
H	0.537020	1.063418	2.548419
H	2.277091	1.192329	2.878737
C	1.798242	-1.357190	2.066562
H	1.995700	-2.259409	1.480544
H	2.571654	-1.278151	2.837495
H	0.824132	-1.486428	2.549242
C	2.507506	0.812044	-2.034406
H	3.538486	0.781215	-2.400624
H	1.832351	0.623143	-2.875983
H	2.310414	1.818298	-1.656110
C	2.660077	-1.637657	-1.516443
H	2.007219	-1.871523	-2.363682
H	3.700132	-1.649067	-1.857405
H	2.545209	-2.432880	-0.772457
O	4.208680	0.292549	0.608963

-1279.095193 A.U.

TS[1]

S	-0.048344	-1.175078	-0.973880
C	-1.359548	-0.285009	-0.251058
C	-2.594207	-1.020163	-0.108903
O	-2.687882	-2.215925	-0.343249
C	-4.852397	-0.996858	0.446090
H	-5.587617	-0.268498	0.791137
H	-5.157281	-1.419513	-0.515550
H	-4.755262	-1.812402	1.168575
O	-3.632457	-0.277783	0.323320
C	-1.091591	1.112028	-0.309918
O	0.061841	1.577879	-0.478286
O	-2.139428	1.933134	-0.222055
C	-1.841237	3.324242	-0.258240
H	-2.793783	3.826794	-0.087398
H	-1.123295	3.591375	0.522552
H	-1.431820	3.610579	-1.230700
C	1.172292	-0.271839	-0.264681
C	1.572458	-0.109200	1.217684
C	2.596461	-0.192820	-0.788600
C	3.019130	-0.156289	0.689759
C	1.267256	1.169392	1.993143
H	1.485245	2.068813	1.416078
H	0.213276	1.191660	2.286041
H	1.884496	1.170264	2.897884
C	1.200600	-1.340151	2.050813
H	1.490294	-2.274353	1.560718
H	1.710937	-1.286168	3.017508
H	0.119063	-1.361240	2.216588
C	2.910910	1.124696	-1.510280
H	3.993602	1.203405	-1.651258
H	2.417532	1.132496	-2.487224

H	2.557940	1.992503	-0.949644
C	3.092580	-1.392413	-1.591220
H	2.621085	-1.417648	-2.579736
H	4.175401	-1.310271	-1.727518
H	2.884368	-2.339652	-1.083533
O	4.087062	-0.178628	1.236522

-1279.057407 A.U.

Thiocarbonyl ylide **6'e**

S	0.028284	-1.062296	0.097500
C	1.513166	-0.230301	0.024453
C	2.582991	-1.232181	-0.046472
O	2.372273	-2.433480	-0.107015
C	4.857025	-1.693846	-0.098476
H	5.787998	-1.126022	-0.088447
H	4.812232	-2.371308	0.758688
H	4.778009	-2.284523	-1.015239
O	3.820864	-0.720583	-0.030651
C	1.614590	1.210286	0.093950
O	0.654166	1.953843	0.240690
O	2.864190	1.685532	-0.018732
C	2.968236	3.100942	0.063317
H	4.032392	3.318666	-0.033143
H	2.406536	3.582582	-0.742311
H	2.589315	3.467297	1.021594
C	-1.398748	-0.277430	0.053841
C	-2.140172	1.049628	-0.105326
C	-2.679179	-1.133763	0.086787
C	-3.413639	0.189745	-0.167904
C	-2.175642	1.958701	1.130913
H	-2.361481	1.389285	2.047712
H	-1.226064	2.484246	1.229174
H	-2.993408	2.676815	1.006297
C	-1.885028	1.837700	-1.389589
H	-1.835654	1.179092	-2.263356
H	-2.713266	2.538830	-1.539451
H	-0.946308	2.387633	-1.305408
C	-3.006041	-1.761690	1.444040
H	-4.041606	-2.117440	1.443547
H	-2.343450	-2.613175	1.634237
H	-2.888547	-1.047040	2.264436
C	-2.838922	-2.146546	-1.046522
H	-2.183574	-3.009560	-0.886319
H	-3.875878	-2.496396	-1.078517
H	-2.595129	-1.706551	-2.018630
O	-4.567303	0.454293	-0.365909

-1279.071049 A.U.

TS^2

S	0.193782	-0.247690	-1.830577

C	-1.081462	0.114083	-0.697938
C	-2.062228	-0.962759	-0.519780
O	-1.957100	-2.050284	-1.064102
C	-4.052922	-1.703051	0.425647
H	-4.800141	-1.317788	1.120154
H	-4.511787	-1.936180	-0.539085
H	-3.592354	-2.612599	0.821470
O	-3.089498	-0.663802	0.290638
C	-1.159811	1.518213	-0.261757
O	-0.447186	2.419869	-0.666030
O	-2.130703	1.728705	0.644060
C	-2.286189	3.086999	1.036585
H	-3.122600	3.093521	1.736401
H	-1.379392	3.459683	1.521871
H	-2.503952	3.721057	0.172905
C	1.147861	-0.181772	-0.441738
C	1.243305	-1.094897	0.785908
C	2.495990	0.480885	-0.234349
C	2.691199	-0.567537	0.876575
C	0.429483	-0.715867	2.025678
H	0.473306	0.356749	2.236042
H	-0.621129	-0.986315	1.885219
H	0.826059	-1.253529	2.893569
C	1.124387	-2.585849	0.472867
H	1.766945	-2.874207	-0.365135
H	1.424454	-3.167387	1.350735
H	0.091778	-2.830453	0.206170
C	2.458129	1.914892	0.311859
H	3.473222	2.203439	0.603195
H	2.080665	2.594797	-0.455384
H	1.806394	2.010783	1.184266
C	3.441914	0.375203	-1.427957
H	3.109518	1.037064	-2.235278
H	4.447927	0.680167	-1.122384
H	3.500386	-0.646939	-1.816304
O	3.636019	-0.889498	1.543084

-1279.046885 A.U.

Thiirane **8e'**

S	-0.092827	-0.466661	1.966492
C	0.720640	-0.057051	0.401043
C	1.619903	-1.187121	-0.048851
O	1.269691	-2.243056	-0.515852
C	3.839633	-1.902924	-0.079435
H	4.809917	-1.497875	0.206798
H	3.618260	-2.810697	0.487472
H	3.818689	-2.135000	-1.147241
O	2.892076	-0.881026	0.227822
C	1.313945	1.331786	0.261630
O	1.278979	2.209107	1.085010
O	1.840787	1.462255	-0.963410

C	2.431259	2.733637	-1.232085
H	2.852986	2.656107	-2.233957
H	1.674974	3.522385	-1.194793
H	3.211593	2.953728	-0.499546
C	-0.768157	-0.171398	0.305969
C	-1.663825	-1.188993	-0.465285
C	-1.787639	0.995524	0.087652
C	-2.729153	-0.088226	-0.451300
C	-1.261961	-1.458419	-1.922655
H	-0.887515	-0.561814	-2.426994
H	-0.486048	-2.223610	-1.965660
H	-2.146971	-1.804743	-2.467549
C	-2.031048	-2.479642	0.245657
H	-2.375865	-2.295255	1.267809
H	-2.835162	-2.983043	-0.301137
H	-1.159655	-3.139691	0.287927
C	-1.425789	2.021444	-0.991639
H	-2.347082	2.480567	-1.365856
H	-0.800790	2.814898	-0.569019
H	-0.896833	1.577639	-1.839721
C	-2.311222	1.700734	1.331183
H	-1.521683	2.312431	1.778966
H	-3.152351	2.344901	1.054313
H	-2.661722	0.989866	2.085460
O	-3.895163	-0.076071	-0.742627

-1279.113489 A.U.

Alkene **5e**

C	0.645379	0.000102	0.000123
C	1.407702	-1.279760	-0.076616
O	1.061203	-2.285262	-0.657240
C	3.320696	-2.397030	0.653377
H	4.189641	-2.170102	1.271080
H	2.758110	-3.234325	1.075040
H	3.628403	-2.655002	-0.363386
O	2.530937	-1.211184	0.652101
C	1.407827	1.279892	0.076665
O	1.061319	2.285656	0.656826
O	2.531100	1.211023	-0.651986
C	3.320950	2.396811	-0.653586
H	4.190056	2.169543	-1.270936
H	2.758547	3.233952	-1.075805
H	3.628378	2.655243	0.363144
C	-0.699708	0.000069	0.000114
C	-1.745164	-1.104231	-0.184694
C	-1.745343	1.104247	0.184906
C	-2.796109	-0.000030	-0.000233
C	-1.824693	-1.652589	-1.616797
H	-1.799664	-0.848797	-2.360847
H	-0.983472	-2.322961	-1.801682
H	-2.769999	-2.192861	-1.735012

C	-1.789924	-2.226108	0.848710
H	-1.700374	-1.840930	1.869951
H	-2.748329	-2.749617	0.766214
H	-0.982038	-2.940819	0.670379
C	-1.789737	2.226174	-0.848496
H	-2.748011	2.749942	-0.766118
H	-0.981701	2.940680	-0.670003
H	-1.700133	1.841023	-1.869740
C	-1.825590	1.652499	1.616974
H	-0.984680	2.323133	1.802288
H	-2.771134	2.192432	1.734841
H	-1.800604	0.848655	2.360971
O	-3.997148	-0.000168	-0.000085

-881.081648 A.U.

Orthorombic sulfur

S	0.001513	2.394767	0.077733
S	1.727571	1.429384	-0.560161
S	2.152693	0.061002	0.919657
S	1.726255	-1.852610	0.099068
S	-0.000960	-1.676668	-0.994917
S	-1.728584	-1.850568	0.098746
S	-2.152513	0.063070	0.919695
S	-1.725974	1.431621	-0.559820

-3184.339450 A.U.
